# A scalable human-zebrafish xenotransplantation model reveals gastrosome-mediated processing of dying neurons by human microglia

**DOI:** 10.1038/s42003-026-09948-6

**Published:** 2026-04-09

**Authors:** Ambra Villani, Jana Wittmann, Tamara Wyss, Izaskun Mallona, Irene Santisteban Ortiz, Nathalie Tichy, Corinna Maria Biermeier, Monique Pena, Ayush Aditya Pal, Darren Gilmour, Simon T. Schafer, Francesca Peri

**Affiliations:** 1 Department of Molecular Life Sciences, University of Zurich, Zurich, Switzerland; 2 SIB, Swiss Institute of Bioinformatics, Zurich, Switzerland; 3 Department of Psychiatry and Psychotherapy, School of Medicine and Health, Technical University of Munich, Munich, Germany; 4 Center for Organoid Systems, Munich Institute for Biomedical Engineering, Technical University of Munich, Garching, Germany

**Keywords:** Cell death and immune response, Apoptosis

## Abstract

Microglia engulf dying neurons through efferocytosis, a critical function in both development and disease. How microglia process the engulfed neuronal material—especially lipids—remains poorly understood, despite its central role in neurodegeneration. Thus, we developed HuZIBRA, a scalable in vivo xenotransplantation model in which human iPSC-derived microglia-like cells (iMGLs) are introduced into the developing zebrafish brain (zf-hiMG), a system characterized by high levels of neuronal cell death and amenable to precise genetic and pharmacological manipulation. We show that human microglia-like cells recognize and engulf apoptotic zebrafish neurons, indicating conserved efferocytic mechanisms. In these cells, engulfed neuronal material accumulates into a distinct, lipid-rich intracellular compartment, the gastrosome, which we also observed in iMGLs placed in a human brain-like environment. The size of the human gastrosome dynamically reflects neuronal cell death levels and is regulated by key genes, including *TREM2* and *SLC37A2*. Pharmacological inhibition of the cholesterol transporter NPC1 induces gastrosome expansion and lipid accumulation, recapitulating pathological features of Niemann-Pick disease type C. Thus, HuZIBRA provides a powerful in vivo platform to uncover cell-autonomous adaptive responses of human microglia to apoptotic and metabolic stress, with the gastrosome emerging as a key integrator of neuronal debris processing and disease-relevant lipid metabolism.

## Abstract (web summary)

Establishment of HuZIBRA, a xenotransplantation model where human iPSC-derived microglia engulf endogenous zebrafish dying neurons, forming a lipid-rich gastrosome regulated by TREM2, SLC37A2 and NPC1, linking neuronal debris processing to disease-relevant lipid metabolism.

## Introduction

Microglia, the resident immune cells of the brain, play a central role in clearing dead neurons through a process known as efferocytosis, the phagocytic clearance of dying cells. This function is essential during brain development and in several neurodegenerative diseases characterized by high levels of neuronal cell death^1–4^. Interestingly, microglia exhibit similar transcriptional profiles in both contexts, suggesting shared functional responses to neuronal cell death^5,6^ (and reviewed in refs. ^7,8^). Efferocytosis—the phagocytic clearance of dying cells—poses unique challenges for microglia during neuronal turnover, requiring efficient degradation of complex neuronal material and careful sorting of byproducts such as lipids. of neurons presents unique challenges for microglia, requiring efficient degradation of neuronal components and sorting of resulting byproducts such as lipids. This process becomes especially critical in disease states, where widespread neuronal death drives microglia to adopt an ameboid morphology often marked by accumulation of lipid inclusions^9–12^. Although the importance of neuronal processing by microglia is increasingly recognized, the underlying cellular mechanisms and the impact of defective processing on microglial shape, plasticity, and function remain largely unknown.

Live imaging studies in zebrafish have shown that microglial phagosomes containing neuronal material fuse with a single compartment called the gastrosome, a post-phagocytic compartment that forms downstream of phagosome maturation^13,14^. This compartment is characterized by a set of unique characteristics, such as an electron-lucent lumen containing membrane fragments and lipids^13^. The gastrosome expands in response to increased neuronal cell death and mutations in *slc37a2*^13^. In phagocytic microglia lacking the NPC1 cholesterol transporter, the gastrosome accumulates gangliosides and cholesterol, driving a shift to an ameboid morphology^14^. This morphological change is further amplified by elevated neuronal cell death, suggesting the gastrosome is an important lipid trafficking compartment highly sensitive to neuronal apoptotic stress.

Given the critical role of microglia in maintaining human brain health, it is essential to determine whether human microglia also possess a gastrosome and how this specialized compartment responds to changes in neuronal cell death. Several studies suggest that genetic differences between animals and humans may lead to variations in microglial behavior and function^15–17^, indicating that animal models may not fully capture the complexity of human microglia biology. To address this, human-induced pluripotent stem cell-derived microglia-like cells (iMGL) have emerged as a valuable model for studying human-specific mechanisms. However, because the brain microenvironment plays a critical role in shaping microglial identity, responsiveness, and dynamics^18,19^, placing human microglia-like cells in a more physiologically representative context has proven essential. Approaches such as colonizing brain organoids with microglia-like cells^20–23^ (and reviewed in refs. ^24,25^) and generating chimeric human/rodent models^17,26–28^ have improved the physiological relevance by providing more accurate brain environments. However, these methods are often time-consuming, resource-intensive, and limited in scalability, which slows experimental throughput and restricts systematic manipulation of microenvironmental factors. Therefore, there is a pressing need to develop scalable approaches that allow precise control over the brain microenvironment, enabling more efficient and detailed investigations into microglial function and their dynamic responses to environmental cues.

In this study, we introduce HuZIBRA (Human Zebrafish Immune-Brain), a scalable in vivo platform for transplanting human iPSC-derived microglia into the zebrafish brain to study their responses to neuronal apoptosis. Live imaging reveals that human microglia complement endogenous microglia and engulf apoptotic neurons, demonstrating high conservation of the efferocytic machinery across species. In these cells, we identify the lipid-rich gastrosome, whose size correlates with neuronal death and is regulated by key genes such as *TREM2* and *SLC37A2*. Inhibiting NPC1 expands the gastrosome and induces lipid accumulation, mimicking Niemann–Pick type C pathology. Thus, HuZIBRA provides a physiologically relevant and scalable system to probe neuronal processing and adaptive responses of human microglia in vivo.

## Results

### A scalable in vivo pipeline for xenotransplantation of human iPSC-derived microglia-like (iMGL) into the zebrafish embryonic brain

**Fig. 1:**
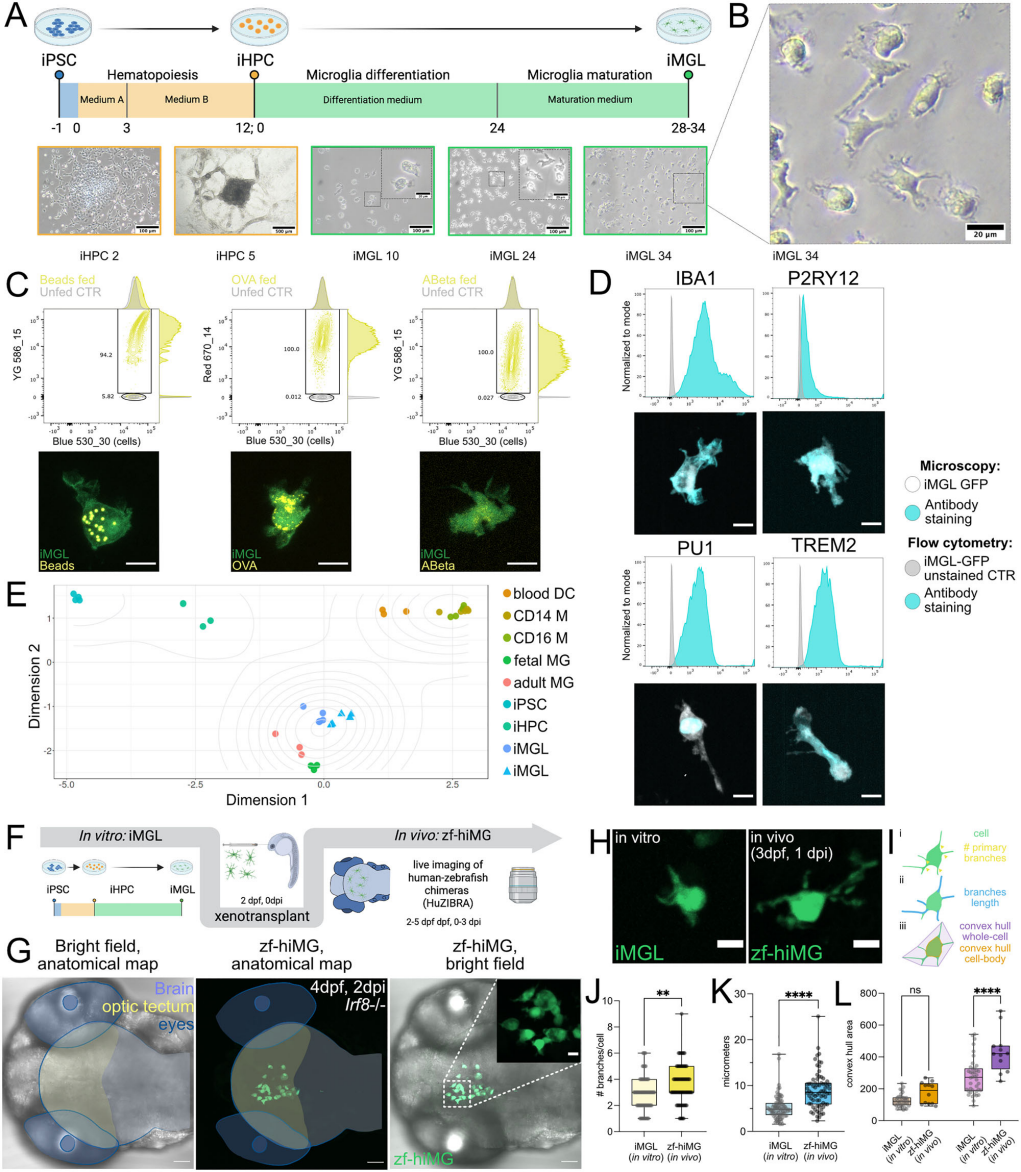
A scalable pipeline for the xenotransplantation of human iPSC-derived microglia (iMGL) into the zebrafish embryonic brain. **A** Schematic overview of the two-step differentiation protocol used to generate hiPSC-derived microglia (iMGL) from fluorescently labeled human iPSCs. **B** Bright field image of WT iMGL in vitro at day 34 of microglia differentiation; scale bar 20 μm. **C** Phagocytic assay in vitro: GFP labeled WT iMGL are fed with fluorescently labeled 1 μm latex beads, ovalbumin or Amyloid-beta and analysed by FACS (upper row) or light microscopy (lower row). Scale bars 10 μm. **D** Immunostaining of microglial markers on GFP-labeled WT iMGL analysed by FACS and light microscopy; scale bars 10 μm. **E** Multidimensional scaling (MDS) comparing WT iMGL obtained in this study (triangles) to iMGL and other cell types obtained by Abud et al. (circles). **F** Schematic of the xenotransplantation protocol of fluorescently labeled iMGL into the embryonic zebrafish optic tectum (zf-hiMG) at 2 days post fertilization (dpf). **G** Representative image of a Irf8st95 zebrafish brain at 4dpf, 2 dpi (days post injection), dorsal view, xenotransplanted with GFP-labeled WT iMGL (zf-hiMG); scale bars 50 μm (overview) and 10 μm (zoomed crop). **H** Comparison of representative cells in vitro (iMGL, left) versus in vivo (zf-hiMG, right); scale bars 10 μm. **I**–**L** Comparison of microglial morphology in vitro (iMGL) versus in vivo (zf-hiMG). Quantification of primary branches (**J**), *p* = 0.0014, unpaired two-tailed Mann–Whitney test; branch length (average per cell) (**K**), *p* < 0.0001, unpaired two-tailed Mann–Whitney test; and convex hull of the cell body or entire cell (**L**) as described in the schematic in (**I**), ns: *p* = 0. 3264, ****: *p* < 0.0001, 2-way ANOVA with Šidàk’s correction for multiple comparisons; N = 3, n = 81(in vitro), n = 74 (in vivo); N = experiments, n = cells. Microscopy data acquired using Andor Dragonfly 200 Sona spinning-disc microscope.

We obtained human iPSC-derived microglia (iMGL) by adopting an established two-step protocol from STEMCELL Technologies (see schematics in Fig. 1A; based on^29^) where green cytoplasmic (WTC-mEGFP-AAVS1-cl6) or red membrane (WTC-mTagRFPT-CAAX-AAVS1-cl91) fluorescently labeled iPSC were first differentiated into hematopoietic progenitors (iHPC), as confirmed by the expression of typical markers for these cells (Supplementary Fig. 1A). Subsequently, iHPC were transferred into microglial differentiation and maturation media (see schematics in Fig. 1A). The resulting induced microglia-like cells (iMGLs) displayed a rudimentary branched morphology (Fig. 1B), phagocytic activity (Fig. 1C) and expressed key microglial markers (Fig. 1D and Supplementary Fig. 1B) Expression of canonical microglial genes in iMGLs was further confirmed both by transcriptome analysis, which showed that these cells cluster together with published human iPSC-derived microglia datasets (^16,17,22,28–30^; Fig. 1E and Supplementary Fig. 1C, D), and by in situ hybridization (HCR), validating mRNA expression of microglial-specific genes in vitro (Supplementary Fig. 1E). These analyses confirmed that iMGLs were transcriptionally distinct from other cell types, such as iHPC precursors and iPSCs (Fig. 1E), and multidimensional scaling revealed strong similarities with both fetal and adult human microglia (Fig. 1E).

To mimic a physiologically relevant brain environment, we transplanted GFP labeled iMGL directly into the optic tectum (OT) of 2-day post-fertilization (dpf) zebrafish embryos, a developmental stage marked by extensive neuronal apoptosis and the establishment of the endogenous microglial population (~25 cells)^31^ (Fig. 1F). The combination of high iMGL culture yields and an efficient injection protocol enabled semi-high-throughput transplantation at a rate of approximately 20 chimeric embryos per operator per hour. We first transplanted iMG into *Irf8*^*st95*^ mutants (also referred to as irf8-/-), which lack endogenous microglia and macrophages^32^ (Fig. 1G). iMG were detected in the OT of approximately 65% of the transplanted embryos (these cells are hereafter referred to as zf-hiMG; Supplementary Fig. 1F, left bar). The number of transplanted zf-hiMG varied, from as few as 1 to over 40 cells per embryo, with an average of 18 (Supplementary Fig. 1G, left bar), highlighting the scalability of the model. We confirmed the survival of zf-hiMG (Supplementary Fig. 1H) and validated their human microglial identity using in vivo Hybridization Chain Reaction analysis (HCR), which demonstrated the expression of key markers, such as *P2Y12*, *IBA1*, and *TREM2* (Supplementary Fig. 1I). Additionally, TREM2 expression was further confirmed also at the protein level (Supplementary Fig. 1J). Together, these data provide evidence that zf-hiMG maintain a typical human microglial identity within the zebrafish brain.

**Fig. 2:**
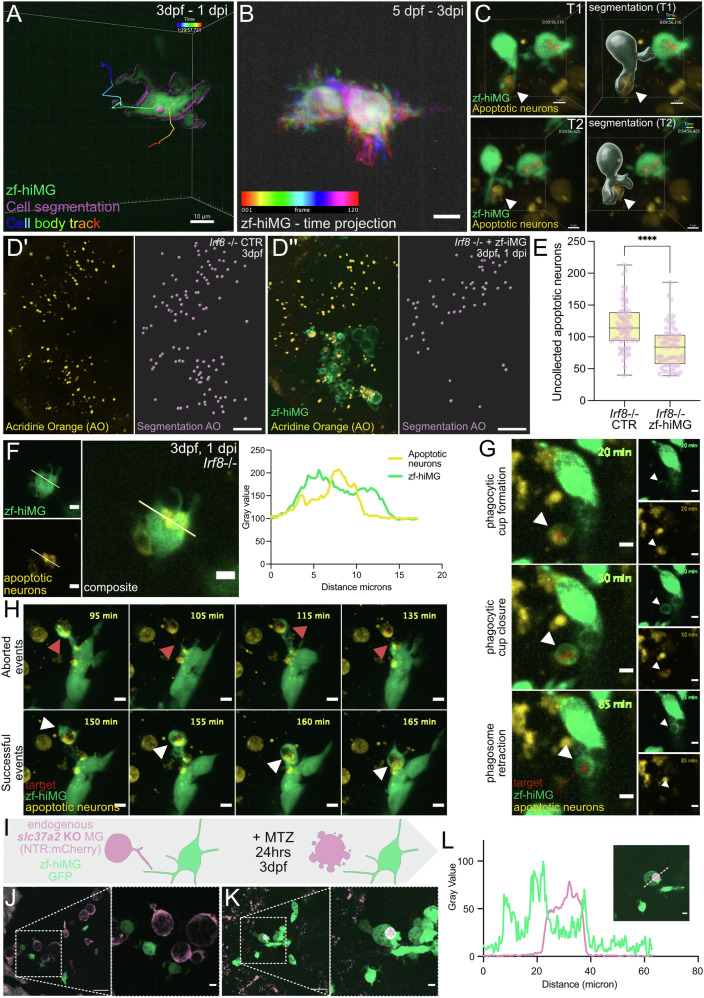
Functional conservation of microglial engulfment mechanisms in a zebrafish–human chimeric model. **A** Segmentation and tracking of GFP-labeled WT zf-hiMG cell body in a 3 dpf, 1 dpi Irf8st95 zebrafish OT; 5 min time resolution; scale bar 10 μm. **B** Time-projection of GFP-labeled WT zf-hiMG in a 5 dpf, 3 dpi zebrafish OT; 1 min time resolution; scale bar 10 μm. **C** Time lapse images showing transient contacts (arrowheads) between GFP-labeled WT zfhiMG branches and nearby apoptotic neurons (Tg(nbt:secA5-BFP)) in Irf8st95 3dpf, 1dpi embryos; 5 min time resolution; scale bar 5 μm. **D** Representative images of Acridine Orange (AO) staining and segmentation in 3 dpf Irf8st95 embryos; (**D**’) control and (**D**”) with membrane RFP labeled WT zf-hiMG transplantation; scale bars 50 μm. **E** Quantification of uncollected AO+apoptotic nuclei in the optic tectum of Irf8st95 embryos ± iMGL transplantation; N = 3, n = 73 (CTR), n = 70 (zf-hiMG), *p* < 0.0001, unpaired two-tailed Mann–Whitney test. **F** Image showing a GFP-labeled WT zf-hiMG transplanted in Irf8st95 containing secA5+ apoptotic neuronal material (Tg(nbt:secA5- BFP)), with quantification of intracellular fluorescent signal; 3 dpf, 1 dpi; scale bar 5 μm. **G** Time-lapse imagies showing GFP-labeled WT zf-hiMG transplanted in Irf8st95 and engulfing an apoptotic neuron (Tg(nbt:secA5-BFP)) by forming a phagocytic cup (arrowhead); track of phagosome retraction in red; 5 min time resolution; scale bar 5 μm. **H** Timelapse images of a GFP-labeled WT zf-hiMG in Irf8st95 attempting sequential engulfment of a dying neuron (Tg(nbt:secA5- BFP)): top, failed phagocytic cup formation attempt and abortion (red arrowhead); bottom, successful re-engagement and engulfment (white arrowhead); 5 min time resolution; scale bars 5 μm. **I** Schematic of metronidazole (MTZ)-induced microglial ablation in slc37a2 KO Tg(fms:Gal4;UAS:nfsB-mCherry) embryos expressing NTR in endogenous zebrafish microglia (MG). Representative images of control **J** and MTZ-treated slc37a2 KO embryos **K** showing selective apoptosis of endogenous NTR+ microglia and uptake of apoptotic material by WT zf-hiMG; scale bars 50 μm (overview) and 10 μm (zoomed crop). **L** Quantification of fluorescent apoptotic signal in zf-hiMG 24 h after MTZ treatment and correspondent qualitative image; scale bar 5 μm. Microscopy data acquired using Bruker Luxendo TruLive3D Imager (**A**–**C**, **F**–**H**, **I**–**L**) and Andor Dragonfly 200 Sona spinning-disc microscope (**D**’, **D**”, **E**).

The small size and optical transparency of zebrafish embryos enabled high spatiotemporal resolution imaging, which we leveraged to investigate the morphology and behavior of transplanted zf-hiMG. To capture these dynamics, we performed whole-brain time-lapse imaging using spinning disk and single-plane illumination microscopy (SPIM)—two high-speed imaging modalities characterized by negligible phototoxicity (Jemielita et al.; Keller and Stelzer). Transplanted cells exhibited a more elaborate branched morphology compared to iMGLs cultured in vitro (Fig. 1H and J), with significantly longer processes (Fig. 1H–I, K) and enhanced motility, marked by continuous cycles of extension and retraction (Supplementary Fig. 1K–L; Fig. 2B; Video 3). Time-lapse imaging further revealed that zf-hiMG were highly dynamic (Fig. 2A; Videos 1–2), suggesting that the zebrafish brain microenvironment supports key microglial properties, including complex morphology and branching dynamics.

To further assess functional engagement with the neural environment, we transplanted iMGL into *Irf8*^*st95*^ mutant zebrafish embryos expressing a fluorescent reporter for apoptotic neurons (Tg(nbt:dLexPR-LexOP:secA5-BFP))^33^. Live imaging revealed transient contacts between zf-hiMG processes and nearby apoptotic neurons (Fig. 2C, video 4), suggesting active sensing of the local microenvironment. Next, we asked whether zf-hiMG could survive and function alongside endogenous zebrafish microglia that we labeled with mCherry. Transplantation efficiency was comparable in zebrafish hosts with or without endogenous microglia (Supplementary Fig. 1F, G), and both populations co-existed within the same brain environment (Supplementary Fig. 2A and video 5).

Taken together, these findings indicate that this newly developed human-zebrafish xenotransplantation platform (HuZIBRA) represents a powerful tool for in vivo exploration of human microglial behavior and cellular interactions within an accessible brain environment.

### Functional conservation of microglial engulfment mechanisms in a zebrafish-human chimeric model

A key function of microglia during development is the engulfment of dying neurons through efferocytosis^34–37^. In *Irf8*^*st95*^ zebrafish mutant embryos, which lack microglia, uncollected apoptotic neurons accumulate in the developing optic tectum (OT)^32^, as visualized by Acridine Orange (AO) staining (Fig. 2D’). Remarkably, transplantation of iMGL into into *Irf8*^*st95*^ embryos significantly reduced the number of uncollected apoptotic nuclei (Fig. 2D’, E). This was further validated using a real-time apoptotic reporter (Tg(nbt:dLexPR-LexOP:secA5-BFP))^33^, which revealed fluorescent apoptotic material inside zf-hiMG vesicles, indicating active uptake of labeled apoptotic neurons (Fig. 2F). Live time-lapse imaging further revealed that zf-hiMG actively engulf endogenous apoptotic neurons by extending branches and forming phagocytic cups around dying cells (Fig. 2G; video 6). These cups either successfully enclosed the target, forming ~5 µm phagosomes that were retracted toward the microglial cell body, or failed to close, resulting in aborted engulfment attempts (Fig. 2H, Video 7), a phenomenon previously observed in zebrafish microglia^33,38^. Notably, failed attempts were often followed by re-engagement and successful engulfment (Fig. 2H, video 7).

To further evaluate the efferocytic capacity of zf-hiMG, we tested their response to induced apoptosis in endogenous microglia. To this aim, we used a genetic system allowing selective ablation of zebrafish microglia (Schematic in Fig. 2I). GFP-labeled iMGL were transplanted into slc37a2-deficient embryos, in which endogenous microglia expressed a nitroreductase–mCherry fusion protein (Tg(fms:Gal4,UAS:nfsB-mCherry)). These slc37a2-deficient microglia are readily identifiable by their abnormally enlarged gastrosome, a consequence of impaired neuronal digestion^13^ (Fig. 2J, SUPP Fig. 2B”, C–C’). Metronidazole (MTZ) treatment triggered Caspase-3-dependent apoptosis specifically in nitroreductase-expressing microglia^31,39–41^ (Supplementary Fig. 2B, compare B” with B”’), but had no effect in non-transgenic controls^31^ (Supplementary Fig. 2C–C’). Within 24 h, red fluorescent apoptotic material was visible inside zf-hiMG (Fig. 2K, L), indicating they could recognize and engulf dying endogenous microglia via conserved “find me” and “eat me” signals. Furthermore, zf-hiMG responded to targeted neuronal laser ablation by polarizing and migrating toward these injury sites (SUPP Fig. 2D, video 8), indicating their capacity to detect and respond to local damage-associated cues.

Finally, we examined whether zf-hiMG could assist endogenous *slc37a2* zebrafish microglia that have an enlarged gastrosome due to neuronal processing defects^13^. iMG transplantation into *slc37a2* mutants led to a marked reduction in gastrosome size within the endogenous *slc37a2* microglia population (Supplementary Fig. 2E–G compare F” with E’, quantification in Supplementary 2G). Because the size of the gastrosome in these mutants is known to depend on the rate of neuronal engulfment^13^, this reduction suggests that zf-hiMG alleviate the burden by clearing apoptotic neurons, thereby functionally complementing the endogenous microglial population.

Together, these results highlight the evolutionary conservation of efferocytic mechanisms between zebrafish and humans and establish this chimeric model as a powerful platform to study human microglial responses to neuronal cell death in a physiologically relevant context.

### Xenotransplantation of human microglia-like cells into zebrafish reveals gastrosome adaptation under phagocytic stress

**Fig. 3:**
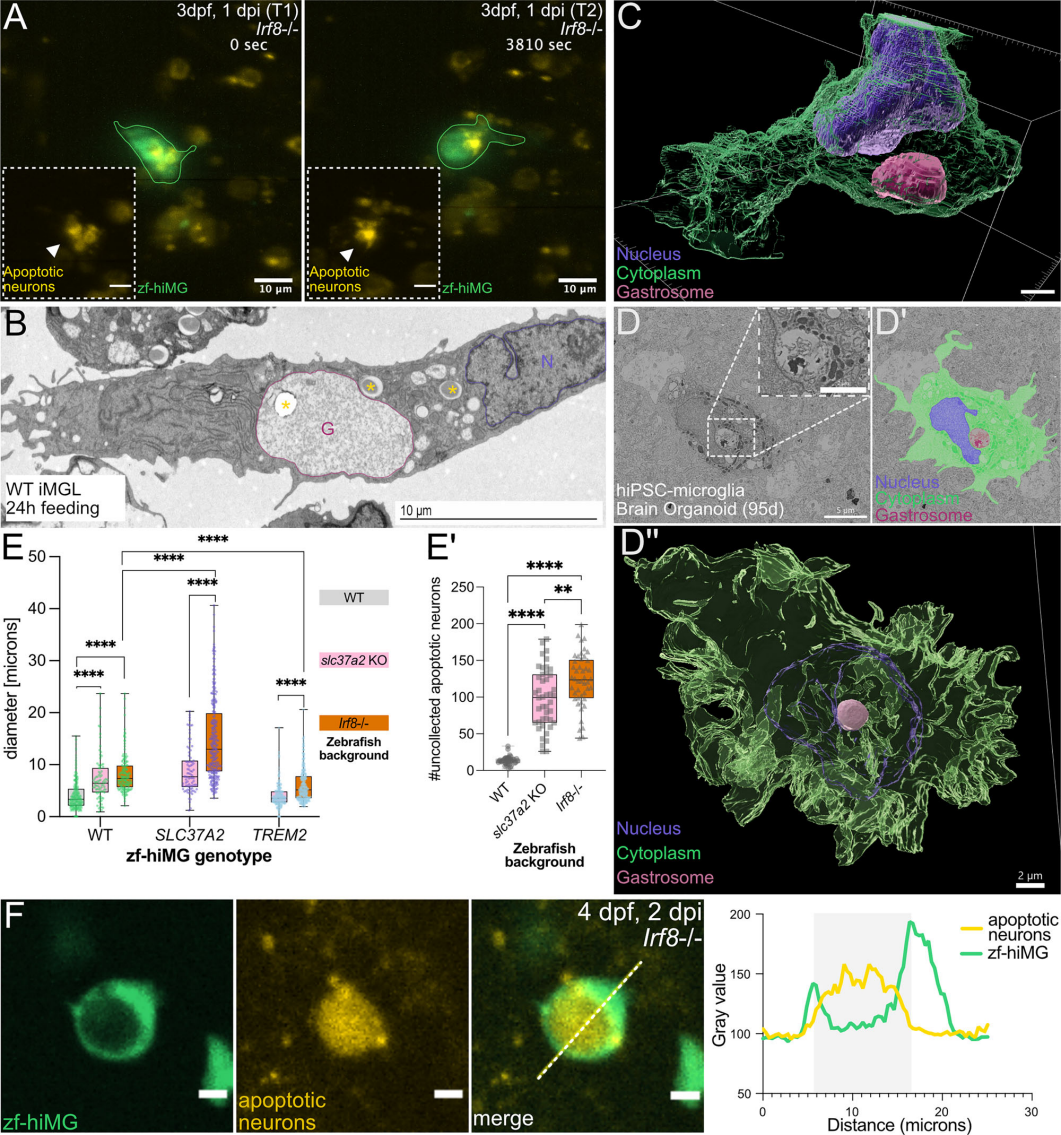
The gastrosome in human microglia-like cells is a conserved, lipid-rich compartment regulated by efferocytic activity. **A** Time-lapse images of GFP-labeled WT zf-hiMG in Irf8^st95^ embryos, showing interaction with neurons marked for apoptosis (Tg(nbt:dLexPR-LexOP:secA5-BFP); 30 s time resolution; 3 dpf, 1 dpi; scale bars 10 μm (overview) and 5 μm (zoomed crop). **B** Electron micrograph (single plane) of 24 h bead-fed WT iMGL in monoculture; N = nucleus, G = gastrosome, * = beads-containing phagosomes; scale bar 10 μm. **C** 3D volume reconstruction of SEM data showing the cytoplasm (green), nucleus (blue), and gastrosome (purple) in WT iMGL in vitro (monoculture); scale bar 2 μm. **D** Single slice from an SEM array of hiPSC-derived microglia integrated into a 95 days old brain organoid; scale bars 5 μm (overview) and 2 μm (zoomed crop); **D**’ segmentation of **D** showing the cytoplasm (green), nucleus (blue) and gastrosome (purple); **D**” 3D volume reconstruction of the segmented cell; scale bar 2 μm. **E** Quantification of the gastrosome’s diameter in GFP-labeled zf-hiMG across different zebrafish backgrounds with increasing apoptotic burden: WT (gray), slc37a2 KO (pink), and Irf8⁻/⁻ (orange). Boxplots show WT zf-hiMG (left column), SLC37A2 zf-hiMG (middle), and TREM2 zf-hiMG (right); N (fish), n (cells) in the table below (data from 5 independent experiments), ****: *p* < 0.0001, Kruskal–Wallis test with Dunn’s correction for multiple comparisons. **E**’ Quantification of uncollected AO^+^ apoptotic nuclei in the optic tectum of wt, slc37a2 and Irf8st95 mutant embryos N = 3, n = 39 (wt), n = 44 (slc37a2 KO) and n = 46 (irf8-/-), **: *p* = 0.001, ****: *p* < 0.0001, 1-way ANOVA with Tukey’s correction for multiple comparisons. **F** Representative image and fluorescence intensity quantification of GFP-labeled WT zf-hiMG in Irf8-/ background with neuronal apoptotic marker (Tg(nbt:dLexPRLexOP: secA5-BFP); 4dpf, 2 dpi; scale bar 5 μm. Microscopy data acquired using Andor Dragonfly 200 Sona spinning-disc microscope (**A**, **E**, **E**’, **F**).

Building on our observations that zf-hiMG can recognize apoptotic zebrafish neurons, we next examined how they process this material. Tracking neuron-derived apoptotic cargo within zf-hiMG revealed progressive intracellular aggregation (Fig. 3A; Video 9), a phenomenon consistent with the presence of the gastrosome—an electron-lucent phagocytic compartment where engulfed apoptotic material accumulates (Villani et al.). To determine whether a similar process occurs in iMGLs, we exposed these cells in culture to fluorescent latex beads and monitored intracellular cargo distribution over a 24-h period. The beads progressively clustered within the cells (Supplementary Fig. 3A), and electron microscopy (EM) revealed the presence of a single enlarged vesicle displaying key features of the gastrosome, including an electron-lucent lumen enclosed by a single membrane and containing membrane fragments^13,14^ (Fig. 3B). While no molecular marker uniquely defines the gastrosome, its ultrastructural characteristics, perinuclear position, size enlargement in response to increased neuronal cell death and lipid content, enable its reliable identification across different contexts^13,14,42^. Given that in vitro cultures inherently experience a certain degree of spontaneous apoptosis and that microglia are likely to engulf these dying cells, some human iMGLs also displayed an enlarged single vesicle in the absence of latex beads (Supplementary Fig. 3B, C). As shown by EM and 3D reconstructions, this compartment shares the same morphological features as the gastrosome observed across different microglial contexts^13,14^ (Fig. 3C and Supplementary Fig. 3D-D’). We next asked whether human microglia that differentiate within a human brain-like environment would also form a gastrosome, pointing to conserved neuronal processing in these cells. Brain organoids provide such a human brain-like environment, and neuronal cell death is an intrinsic feature of their growth and maturation^23^. Indeed, we have previously shown that long-term organoid culture leads to necrotic core formation and the emergence of developmentally stressful microenvironments^23^. Consistent with this, EM imaging and 3D segmentation of enlarged ameboid microglia within organoids revealed a single, perinuclear, electron-lucent vesicle containing cellular debris, further supporting the presence of a gastrosome-like compartment in human microglia under these conditions (Fig. 3D-D” and Video 10).

As high levels of neuronal apoptosis is a hallmark of many human disease contexts, including neurodegenerative disorders and brain injuries, we next examined how the microglial gastrosome responds to apoptotic stress and increased engulfment demands. To this end, we transplanted WT zf-hiMG into three different zebrafish models characterized by different levels of apoptosis: WT (low burden), *slc37a2* mutants (moderate burden)^13^, and *Irf8*^*st95*^ (high burden)^32^ (Fig. 3E’ and Supplementary Fig. 3F). Gastrosome size increased progressively across these conditions, correlating with the apoptotic load (Fig. 3E, leftmost column: gray, pink, and orange boxplots for WT zf-hiMG). Imaging in the *Irf8*^*st95*^ background, combined with a marker for apoptotic neurons, confirmed that enlarged gastrosomes contained neuron-derived material (Fig. 3F). The dose-dependent enlargement underscores the gastrosome’s adaptive responsiveness to increasing levels of apoptotic stress, further supporting its role as a specialized phagocytic compartment for processing engulfed cellular debris.

**Fig. 4:**
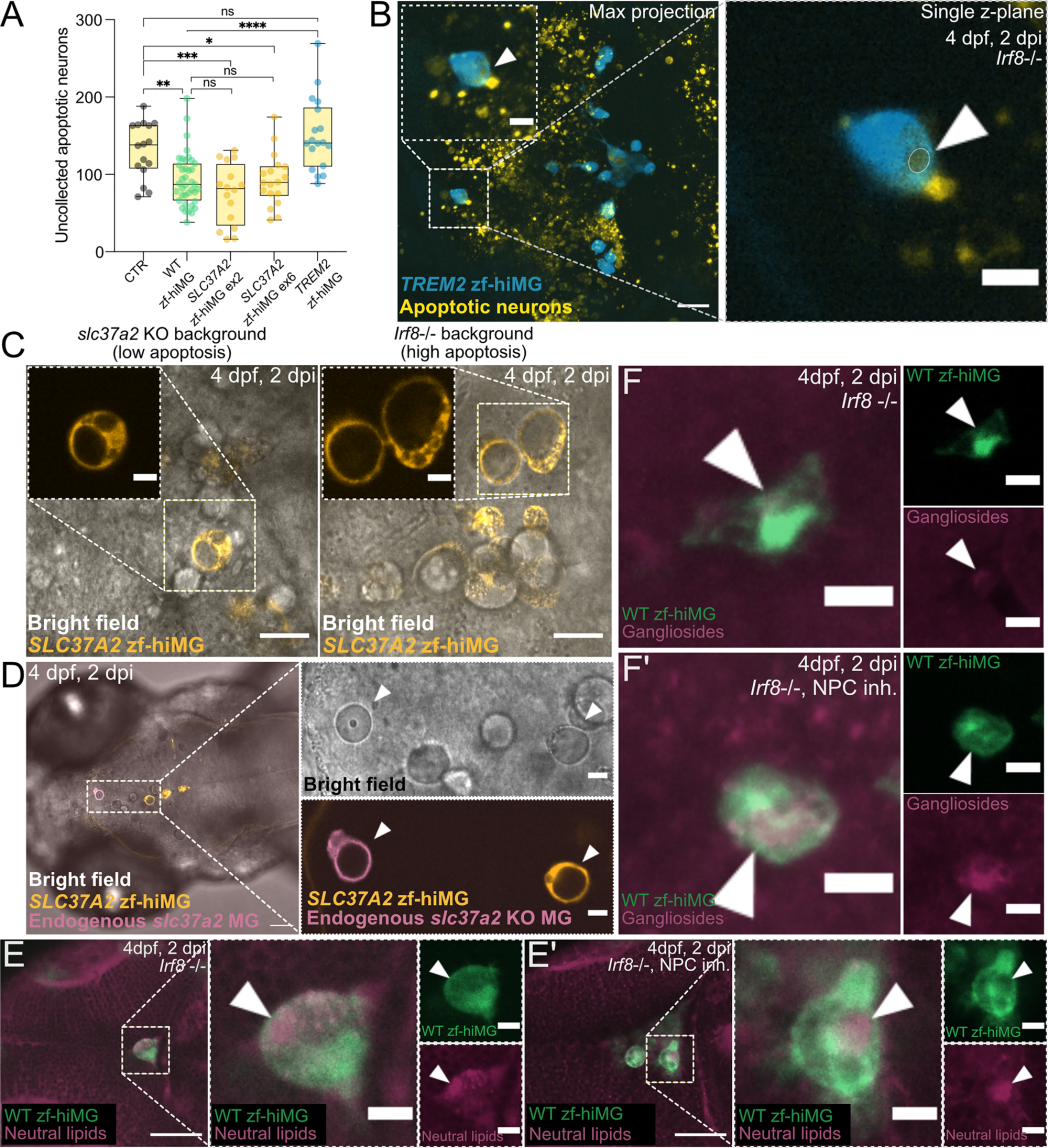
Genetic and functional perturbations alter gastrosome dynamics and lipid accumulation in human microglia-like cells in vivo*.* **A** Quantification of uncollected Acridine Orange (AO) positive apoptotic neurons in Irf8st95 embryos without (CTR, gray dots) nor with transplantation of WT (green dots), SLC37A2 mutant (orange dots, exon 2 and exon 6 clones) or TREM2 mutant (blue dots) zf-hiMG; 3 dpf, 1dpi; N = 6 independent experiments, n (embryos) = 16 (CTR), 39 (WT zf-hiMG), 16 (SLC37A2 ex2 zf-hiMG), 18 (SLC37A2 ex6 zf-hiMG), 18 (TREM2 zf-hiMG); ns (CRT vs. TREM2 zf-hiMG): *p* = 0.6549, ns (WT zf-hiMG vs. SLC37A2 ex2 zf-hiMG): *p* = 0.5583, ns (WT zf-iMG vs. SLC37A2 ex6 zf-hiMG): *p* > 0.9999, *: *p* = 0.0213, **: *p* = 0.0049, ***: *p* = 0.0003, ****: *p* < 0.0001, 1-way ANOVA with Šidák’s correction for multiple comparisons. **B** Representative image of GFP-labeled TREM2 zf-hiMG in Irf8st95 embryos with neuronal apoptotic marker (Tg(nbt:dLexPR-LexOP:secA5-BFP), arrowhead pointing at the largest vesicle in the cell; dorsal view; scale bars 30 μm (overview) and 10 μm (zoomed crop). **C** Bright field and fluorescence representative images of SLC37A2 zf-hiMG in Irf8st95 and slc37a2 KO backgrounds; dorsal view, 4dpf, 2 dpi; scale bars 30 μm (overview) and 10 μm (zoomed crop). **D** GFP-labeled SLC37A2 zf-hiMG into slc37a2 KO embryos with red-labeled endogenous microglia; dorsal view, 4dpf, 2 dpi; scale bars 30 μm (overview) and 10 μm (zoomed crop). Neutral lipid staining (HCS LipidTox) in xenotransplanted GFP-labeled WT zf-hiMG in Irf8st95; with (**E’**) or without (**E**) pharmacological inhibition of NPC1; dorsal view; 4 dpf, 2 dpi; scale bars 50 μm (overview) and 10 μm (zoomed crop). **F** Staining with fluorescently labelledn cholera toxin B (CtxB) to visualize GM1 gangliosides in GFP labeled WT zf-hiMG transplanted into Irf8st95 with (**F**′) or without (**F**) NPC1 inhibition; 4 dpf, 2 dpi; dorsal view; scale bars 10 μm. Microscopy data acquired using Andor Dragonfly 200 Sona spinning-disc microscope.

Given the observed correlation between gastrosome size and neuronal cell death^13,14^, we next asked whether this compartment’s responsiveness would be altered in microglia with impaired phagocytic function. TREM2, a key regulator of microglial phagocytic functions and a well-established risk factor in multiple neurodegenerative diseases^5,30,43,44^, appeared as a suitable candidate to test this hypothesis. To this end, we generated *TREM2*-deficient hiPSC using CRISPR-Cas9, targeting exon 2 with a previously published sgRNA^44^ (Supplementary Fig. 4A). We obtained a clone carrying a homozygous small deletion (Supplementary Fig. 4A’), which led to significantly reduced transcript levels as confirmed by RNA-seq (Supplementary Fig. 4B). The edited cells retained pluripotency (data not shown) and differentiated efficiently into iMGL (*TREM2* iMGL) expressing canonical microglial markers at levels comparable to WT iMGL (Supplementary Fig. 4B). Transplantation of *TREM2* zf-hiMG into *Irf8*^*st95*^ embryos resulted in a significantly higher number of uncollected apoptotic neurons compared to embryos transplanted with WT zf-hiMG (Fig. 4A). Notably, the number of unengulfed apoptotic neurons was comparable to that observed in non-transplanted *Irf8*^*st95*^ embryos, indicating that *TREM2*-deficient zf-hiMG fail to compensate for the absence of endogenous zebrafish microglia. This functional impairment is consistent with previous studies in other systems^45–50^. In line with this, *TREM2* zf-hiMG in the *Irf8*^*st95*^ background displayed a significantly smaller gastrosome compared to WT zf-hiMG in the same apoptotic context (Fig. 3E, orange boxplots, rightmost vs. leftmost column; Fig. 4B for a representative example). When transplanted into *slc37a2* mutant embryos, which retain endogenous microglia, *TREM2* zf-hiMG showed an even further reduction in gastrosome size, likely reflecting reduced access to apoptotic material due to competitive engulfment (Fig. 3E, rightmost column, compare orange to pink boxplot). Despite their diminished phagocytic performance, *TREM2* zf-hiMG retained dynamic morphology and active scanning behavior, indicating that they remained viable and responsive in vivo (Supplementary Fig. 4D, Video 11). Together, these findings demonstrate that the gastrosome in human microglia-like cells adapts to the rate of neuronal cell death and is sensitive to genetic perturbations in efferocytosis pathways. The observed reduction in gastrosome size in TRME2-deficient cells further underscores the role of this compartment in neuronal clearance and establishes the gastrosome as a conserved, functionally responsive structure central to microglial phagocytic competence.

### In vivo characterization of the gastrosome in human microglia-like cells reveals a conserved role in lipids handling

To further investigate the gastrosome in human microglia, we next focused on its regulation and size. Given that *SLC37A2* has been implicated as a critical regulator of the gastrosome^13,14^, we used CRISPR-Cas9 genome editing to generate *SLC37A2*-deficient human iPSCs by targeting either exon 2 or exon 6 (Supplementary Fig. 4C). Both resulting clones harbored indels confirmed by sequencing (Supplementary Fig. 4C’) and showed a reduction in transcript levels by RNA-seq (Supplementary Fig. 4B). Pluripotency was retained in hiPSCs (data not shown) and the mutant cells were successfully differentiated into iMGL (*SLC37A2* iMGL) expressing canonical microglial markers at levels comparable to WT iMGL (Supplementary Fig. 4B). We transplanted *SLC37A2-*deficient iMGL into *Irf8*^*st95*^ embryos and labeled apoptotic neurons using AO. Quantification revealed that both xenotransplanted *SLC37A2-deficient* lines significantly reduced apoptotic burden compared to non-transplanted controls, reaching levels similar to those achieved by WT zf-hiMG (Fig. 4A). However, despite this preserved engulfment capacity, *SLC37A2-deficient* zf-hiMG showed a markedly enlarged gastrosome 48 h post transplantation compared to WT zf-hiMG in the same background (Fig. 3E, compare orange boxplots in middle vs. left columns; Fig. 4C for representative images), indicating a defect in downstream processing of engulfed material. To further investigate this phenotype, we transplanted green-labeled *SLC37A2-deficient* iMGL into *slc37a2* mutant embryos, in which endogenous microglia were labeled with mCherry. Two days post-transplantation, human and zebrafish *SLC37A2*-deficient microglia exhibited highly similar morphologies, appearing nearly indistinguishable (Fig. 4D). Interestingly, the gastrosome size in *SLC37A2-deficient* zf-hiMG was smaller in *slc37a2* mutant embryos than in Irf8^st95^ embryos, likely reflecting reduced availability of apoptotic substrates due to the presence of resident microglia (Fig. 3E, middle column; compare pink vs. orange boxplots; Fig. 4C).

To assess the biochemical content of the enlarged gastrosome, we stained WT and *SLC37A2-deficient* zf-hiMG in the *Irf8*^*st95*^ mutant background with cholera toxin B (CtxB), a marker for GM1 ganglioside. This revealed an accumulation of GM1 gangliosides within the enlarged gastrosome of *SLC37A2-deficient* zf-hiMG, indicating lipid buildup (Fig. 4F and Supplementary Fig. 4E). Lipid accumulation is a well-described feature of microglia in neurodegenerative disease contexts and is thought to reflect impaired degradation or trafficking of lipid-rich neuronal debris^5,6,9–12,14^. To test whether gastrosomal expansion reflects a general response to disrupted lipid trafficking, we transplanted human iMGL into *Irf8*^*st95*^ embryos and exposed these embryos to U18666A, a well-characterized inhibitor of NPC1—a cholesterol transporter whose mutation causes Niemann-Pick disease type C^51^. NPC1 is essential for proper microglial function, and microglial somatic enlargement has been reported in this disease context^14,52^. Upon NPC1 inhibition, we observed a robust expansion of the gastrosome in zf-hiMG mirroring the response of endogenous microglia within the same embryo (Supplementary Fig. 4F-F’). Staining with LipidTOX and CtxB consistently revealed accumulation of neutral lipids and gangliosides within the enlarged gastrosome (Fig. 4E-E’ for neutral lipids and Fig. 4F-F’ for gangliosides). While precise quantification was technically challenging due to variability in dye penetration and signal intensity in vivo, these qualitative readouts robustly indicate lipid and ganglioside enrichment following NPC1 inhibition, as previously observed in zebrafish microglia and human fibroblasts^14^.

Together, these results demonstrate that the gastrosome is a conserved and functionally responsive compartment in human microglia. Its expansion in response to genetic and pharmacological disruption of lipid processing highlights the gastrosome’s role in the degradation and clearance of lipid-rich neuronal debris—a function critical for sustaining microglial homeostasis in both healthy and disease-associated states.

## Discussion

Impaired phagocytosis and disrupted lipid processing in microglia are increasingly recognized as contributing factors in the pathogenesis of neurodegenerative diseases^5,11^. To investigate how human microglia process neuron-derived material, iPSC-derived microglia (iMGL) offer a powerful tool, providing a human genetic background and avoiding species-specific differences in gene regulation and behavior. However, to accurately capture microglial function, these cells must be studied in environments that mimic native tissue architecture and cues. Rodent xenotransplantation models and brain organoids provide valuable complexity and physiological relevance, but they are also time-consuming, technically demanding, and limited in throughput. In this context, HuZIBRA offers a complementary approach. Transplantation of iMGL into the developing zebrafish brain is technically simple, scalable, as it allows the generation of multiple biological replicates efficiently. Zebrafish injections are rapid, and their short breeding cycles enable the expansion of experimental sample sizes within days rather than months, making the system well suited for high-throughput or comparative studies. The workflow presented here is inherently species-agnostic, and the same transplantation, imaging, and analysis pipeline could be readily applied to mouse cells. The main practical limitation is the requirement for relatively high cell numbers, which are more easily obtained from immortalized lines than from primary cultures.

While questions related to long-term integration of human microglia may be better suited to organoid or rodent systems^20–23^, HuZIBRA can help with capturing acute, cell-autonomous microglial responses to neuronal apoptotic and metabolic stress. The zebrafish brain provides a permissive, in vivo environment in which human microglia exhibit highly branched morphologies and significantly more dynamic behavior than in vitro. Although some behavioral features may be dependent on the specific zebrafish context, the model’s small size and optical transparency enable high-temporal-resolution imaging of human microglia in real time. This reduces the risk of underestimating cell speed or oversimplifying migratory trajectories—limitations commonly encountered in slower or lower-resolution imaging platforms. Within HuZIBRA, human microglia are observed making transient, repeated contacts with dying zebrafish neurons. Such interactions resemble behaviors reported for endogenous zebrafish microglia^33,38^, and echo transient synaptic contacts observed in rodent models^53^ (for review on the topic see ref. ^54^). Together, these findings point to a conserved feature of microglial surveillance and efferocytosis across species, highlighting HuZIBRA as a powerful tool for investigating core aspects of human microglial behavior in vivo. While the HuZIBRA model offers clear advantages in terms of scalability, accessibility, and imaging resolution, it also has some limitations. In particular, observations are typically limited to the first 3 days post-transplantation, when the embryos remain optically transparent—a key factor that dictates the feasible imaging window. Since human microglia do not proliferate during this time window, the model is less suited for applications that require large amounts of material, such as bulk or single-cell omics. However, this limitation is likely to become less relevant with the continued advancement of spatial transcriptomics—a rapidly evolving field to which HuZIBRA is well positioned to contribute in the future.

A key outcome of this study is the demonstration of conserved microglial efferocytosis across species. When transplanted into the zebrafish brain, human iPSC-derived microglia effectively recognize and engulf dying zebrafish neurons, indicating that core molecular cues guiding this process are functionally preserved. Although we cannot formally exclude engulfment of viable neurons, all observed phagosomes contained SecA5-apoptotic positive material, indicating uptake of apoptotic neurons. This cross-species compatibility provides a unique opportunity to model human microglial responses to neuronal cell death in vivo, and to dissect how genetic or pharmacological perturbations influence their clearance behavior. These dynamic processes are often understudied, partly due to the technical difficulty in imaging highly dynamic processes deep in the brain. Yet, they are highly relevant to understand how microglia respond to high levels of neuronal apoptosis that are typical of development and neurodegenerative conditions. To date, omics approaches have identified key gene expression programs and regulatory networks; however, our understanding of how these programs translate into core cellular processes, such as debris clearance, lipid metabolism, and dynamic environmental responses, remains limited. In this study, we observed that human microglia process apoptotic neuronal material through a coordinated pathway that involves the lipid-rich gastrosome. This compartment expands in response to increased neuronal death and is significantly reduced in microglia lacking functional TREM2, a receptor linked to neurodegenerative disease. These findings reveal gastrosome-mediated processing of neuronal material by human microglia in vivo and position HuZIBRA as a powerful, real-time system to probe both cell-intrinsic and microenvironmental control of microglial behavior.

## Materials and methods

### Animal model

Zebrafish (Danio rerio) were raised, maintained, and bred according to standard procedures as described in “Zebrafish—A practical approach” (Nüsslein-Volhard, 2012). All experiments were performed in the Tubingen and golden background on embryos younger than 5 dpf, in accordance with the European Union Directive 2010/62/EU and local authorities (Kantonales Veterinäramt; Fishroom licence TVHa Nr. 178). Live embryos were kept at 28 °C in E3 solution, and staging was done according to ref. ^55^. In this study, sex determination is not relevant since in zebrafish, all individuals develop initially immature ovaries, and the process of gonadal differentiation and sex determination takes place around 25 days post-fertilization^56^. To avoid pigmentation, 0.003% 1-phenyl-2-thiourea was added at 1dpf. The following lines were used in this study *irf8*^*st95*^^32^, *slc37a2*^*NY007* 13^, Tg(mpeg1:GFP-caax)^13^, Tg(nbt:dLexPR-LexOP:secA5-BFP)^33^, TgBAC(fms:Gal4,UAS:nfsB-mCherry)^57^, the Tg(UAS:lyn-miRFP670) plasmid was obtained by cloning the lyn-miRFP670 fusion protein downstream of the UAS promoter using the Gateway cloning kit (Thermo Fisher Scientific). This construct, flanked by Tol2 sites, was injected into 1-cell-stage Tg(fms:Gal4) embryos to generate transgenic lines expressing membrane-labeled, far-red microglia. We have complied with all relevant ethical regulations for animal use.

### Maintenance of iPSC and generation of fluorescent reporter lines

For zebrafish transplantation experiments and iMGL differentiations, we used the following labeled human induced pluripotent stem cells (iPSC) lines: AICS-0036-006 (WTC-mEGFP-Safeharborlocus(AAVS1)-cl6(mono-allelic tag)) and AICS-0054-091 (WTC-mTagRFPT-CAAX-Safeharborlocus(AAVS1)-cl91 (mono-allelic tag)), which were developed at the Allen Institute for Cell Science (allencell.org/cell-catalog) and available through Coriell. The lines were cultured in mTeSR1 or mTeSR Plus (STEM CELL Technologies) in a humified incubator at 5% CO2, 37 °C by following the guidelines provided by STEMCELL Technologies. For organoid-microglia co-culture experiments, the following neurotypical control iPSC lines were used: Cent 3-6, Clue4-7. The iPSC lines used in this study have previously been described (Schafer et al., Nat Neuroscience), and the protocol for the use of these human pluripotent stem cell lines was approved by the Ethics Committee of the Technical University of Munich (#2022-250-S-KH and #2024-72-S-DFG-CB). hiPSC cultures were maintained in iPS-Brew media (Miltenyi Biotec) under feeder-free conditions using Matrigel-coated plates Sarstedt). All cell lines were regularly tested for mycoplasma contamination. To generate the tdTomato-positive fluorescent reporter lines for the organoid co-culture experiments, we infected iPSCs with LV-CAG::tdT^23^. Five days after infection, cells were isolated using FACS and re-plated on Matrigel-coated plates in iPS-Brew media (Miltenyi) supplemented with CloneR (Stem Cell Technologies). Upon recovery, cell lines were expanded and used for subsequent experiments.

### CRISPR-mediated editing of SLC37A2 and TREM2

gRNA targeting the second exon of *TREM2* (5′-CTCTCCCAGCTGGCGGCACC-3′)^44^, or the second (5′-CTATCAGTATCGTCAAGGTG-3′) or sixth (5′-AATGGACTCGTCCAGACCAC-3′) exons of *SLC37A2* were designed using the CRISPR/Cas9 target online predictor CHOPCHOP^58^ and ordered from IDT as crRNA (Table S1). The RNP complex was obtained following the manufacturer guidelines (IDT). Human iPSC were cultured in StemFlex Medium (ThermoFisher), and the RNP complex was introduced by electroporation (Lonza). Cells were diluted and seeded as single cells to obtain monoclonal lines using the isoCell-isoHub system (iota biosciences, Alameda, US) by following the manufacturer guidelines. Successful targeting was confirmed by PCR and sequencing using primers listed in Table S2.

### Differentiation of iPSC-microglia (iMGL) from iPSCs

Human iPSC were differentiated to hemotopoietic progenitor cells (iHPC) using the Hematopoietic Kit (STEMCELL Technologies) and then used to further differentiate mature induced microglia-like cells (iMGL) using the Microglia Differentiation and Microglia Maturation kits (STEMCELL Technologies) by following the manufacturer guidelines.

### Generation of forebrain organoids

The above indicated subject-derived iPSC lines (Cent 3-6, Clue4-7) were used to generate forebrain organoids as described previously with minor modifications^59,60^. Human iPSC colonies were detached before reaching confluency with collagenase Type IV (Gibco) and transferred to an Ultra-Low attachment 10-cm plate (Corning Costar) containing 10 ml hPSC medium consisting of DMEM:F12 (Invitrogen), 20% Knockout Serum Replacement (Gibco), 1 × Non-essential Amino Acids (Invitrogen), 1 × 2-mercaptoethanol (Gibco), 1 × GlutaMAX (Invitrogen), 10 ng ml^–1^ FGF-2 (Peprotech) and ROCK inhibitor Y27632 (10 μM). Twenty-four hours later, the medium was replaced with induction medium containing hPSC media without FGF-2, 2 μM dorsomorphin (Tocris), and 2 μM A-083 (Tocris). At day 5 the media was replaced with neural induction medium consisting of DMEM:F12 (Invitrogen), 1 × N2 Supplement (Invitrogen), 1 × Non-essential Amino Acids (Invitrogen), 1 × GlutaMAX (Invitrogen), 10 μg ml^–1^ Heparin (Tocris), 1 × Penicillin/Streptomycin (Gibco), 10 μM CHIR99021 (Tocris), and 1 μM SB-431542 (Tocris). Seven days after induction, organoids were embedded in 20-μl Matrigel (CultrexTM, Bio-Techne) droplets and continued to grow for an additional week in 6 cm Ultra-Low attachment plates (Corning Costar). From day 14 onwards, organoids were cultured in differentiation medium consisting of DMEM:F12 (Invitrogen), 1 × N2 and B27 Supplements (Invitrogen), 1 × Non-essential Amino Acids (Invitrogen), 1 × GlutaMAX (Invitrogen), 1 × 2-Mercaptoethanol (Gibco), 1 × Penicillin/Streptomycin (Gibco), and 2.5 μg ml^–1^ Insulin (Sigma), and transferred to an orbital shaker (65–75 rpm). At day 20, residual Matrigel was removed, and media changes were performed every 2–3 days using the aforementioned differentiation medium. At day 42, forebrain organoids were prepared for subsequent colonization with erythromyeloid progenitor cells (see section below).

### Colonization of cortical forebrain organoids through erythromyeloid progenitors (EMPs)

EMPs were generated as previously described with minor modifications^29^ using the iPSC lines indicted above (Cent 3-6, Clue4-7). Briefly, iPSCs were dissociated using TrypLE (Invitrogen) and plated in a 6-cm tissue culture-treated plate (CytoOne USA Scientific) at a density of 400,000 cells per well with 10 μM ROCK inhibitor (Tocris). The next day, cells were changed to basal hematopoietic differentiation media supplemented with FGF2 (50 ng ml^−1^, Miltenyi), BMP4 (50 ng ml^−1^, Proteintech), Activin A (12.5 ng ml^−1^, Miltenyi), ROCK inhibitor (1 μM, Tocris), and LiCl (2 mM, Sigma) and grown under hypoxic conditions (5% CO2, 5% O2). Hematopoietic differentiation media consisted of IMDM (50%, Thermo Fisher Scientific), DMEM/F12 (50%), ITSG-X, (2% v/v, Thermo Fisher Scientific), L-ascorbic acid 2-Phosphate magnesium (64 mg ml^−1^; Sigma), monothioglycerol (400 mM), PVA (10 mg ml^−1^; Sigma), Glutamax (1×, Thermo Fisher Scientific), chemically defined lipid concentrate (1×, Thermo Fisher Scientific), non-essential amino acids (NEAA, Thermo Fisher Scientific 1×), Penicillin/Streptomycin (P/S Thermo Fisher Scientific, 1% V/V). On day 2, media was changed and supplemented with FGF2 (50 ng ml^−1^, Miltenyi) and VEGF (50 ng ml^−1^, Miltenyi) and returned to hypoxic conditions. Following day 4, cells were placed into norm-oxic conditions (5% CO2, 21% O2) and kept in basal hematopoietic differentiation media supplemented with FGF2 (50 ng ml^−1^, Tocris), VEGF (50 ng ml^−1^, Miltenyi), TPO (50 ng ml^−1^, Miltenyi), SCF (10 ng ml^−1^, Miltenyi), IL-6 (50 ng ml^-1^, Miltenyi), and IL-3 (10 ng ml^−1^, Miltenyi) until colonies released hematopoietic stem cells (usually between day 14 and 16). EMPs were then isolated using Fluorescence-activated cell sorting (FACS) by gating on tdT- and CD43-FITC (Biolegend, 315204)-positive cells. Co-culture of isolated EMPs with cortical organoids was performed at 42 days of forebrain organoid differentiation using 100,000 cells in the above-indicated organoid differentiation media supplemented with 25 ng ml^−1^ M-CSF (Miltenyi), 100 ng ml^−1^ IL34 (Miltenyi), and 50 ng ml^−1^ TGFβ1 (Miltenyi). The organoid-resident EMPs were allowed to differentiate into microglia-like cells for up to 4 weeks post integration using the above indicated media.

Organoid were imaged using a Leica THUNDER microscope equipped with a Leica K5 camera (Leica Microsystems, Wetzlar, Germany) and an environmentally controlled imaging chamber (Okolab, Pozzuoli, Italy).

### Flow cytometry

On day 12 of iHPC differentiation, hematopoietic markers were tested via antibody staining (see Key Resource Table) and FACS analysis. 5*10^4^–2*10^5^ cells/sample were incubated with antibodies diluted in FACS Buffer (D-PBS without Mg^++^ and Ca^++^ with 2% FBS) for 30 min at 4 °C. The cells were washed twice with FACS buffer and resuspended in 300 µl sorting buffer (FACS buffer with 1 mM EDTA and 25 mM HEPES) for analysis. On day 24 of iMGL differentiation, microglial markers were tested via antibody staining and FACS analysis. 5*10^4^–2*10^5^ cells/sample were resuspended in 100 µl of FACS buffer and blocked with 100 µl of 10 µg/ml CD32 (20 min at 4 °C). The samples were then incubated 45 min at 4 °C with the primary antibodies (see list below), and then 20 min at room temperature with secondary antibodies. The cells were washed twice with FACS buffer and then resuspended in 300 µl sorting buffer for analysis. The samples were analyzed at a BD LSR II Fortessa Analyzer and processed in FlowJo.

### Phagocytosis assay in vitro

Mature iMGL seeded at a density of 1*10^5^ cells/cm^2^ on Matrigel-coated cell culture vessels were exposed to fluorescent Amyloid-β (Aβ) 1-42 (0.55 µg/cm^2^), ovalbumin (4.21 µg/cm^2^), or 1 µm large latex beads (diluted in a 1:5 ratio in not heat-inactivated FBS). 12–24 h after the feeding, the cells were analyzed either by FACS or light microscopy.

### Staining procedures

#### HCR RNA-FISH in vitro and in vivo

The mRNA of selected targets was stained by HCR RNA-FISH (Molecular Instruments). HCR buffers, reagents, hairpins, and probes were obtained from the manufacturer. The target mRNA sequences for the human-specific HCR probes were based on the MANE select transcript variants available on NCBI (Table 2S3). HCR probes were designed to reduce cross-reactivity between zebrafish and human genes (Table 5S4). For in vitro HCR experiments, mature iMGL were fixed by first adding 8% PFA directly to the culture medium to achieve a final concentration of 4% PFA (20 min at room temperature), followed by replacement with fresh 4% PFA in PBS, which was incubated for 10 min at room temperature. HCR was then performed by following the manufacturer guidelines for mammalian cells on a chambered slide. For in vivo HCR experiments, 4 dpf zebrafish embryos were fixed overnight in PFA 4% at 4 °C and dehydrated and permeabilized as by manufacturer guidelines with a proteinase K incubation of 30 min (protocol for whole-mount zebrafish embryos and larvae).

#### Immunohistochemistry in cell culture

Mature iMGL were fixed by first adding 8% PFA directly to the culture medium to achieve a final concentration of 4% PFA (10–20 min at room temperature), followed by replacement with fresh 4% PFA in PBS, which was incubated for 20 min at room temperature or overnight at 4 °C. The cells were then rinsed in PBS and permeabilized for 7 min at room temperature (0.2% Triton-X-100/PBS). After blocking for 1 h at room temperature (3%BSA/0.2%Triton-X-100/PBS), the cells were incubated with the primary antibody diluted in blocking solution overnight at 4 °C. The cells were rinsed 3 times with PBS1× and incubated 1 h at room temperature with the secondary antibody and DAPI in blocking solution.

#### AnnexinV staining

A red labeled AnnexinV detection kit (ThermoFisher) was used to detect apoptosis in vitro by following the manufacturer guidelines. The samples were then analyzed by FACS. To analyse the staining by light microscopy, 3 µl of AnnexinV conjugate were added directly to a well of mature iMGL which were immediately imaged by spinning disk microscopy.

#### Acridine orange

To visualize apoptotic cells in vivo, 3 dpf control and transplanted embryos were stained with acridine orange (AO, Merck, Sigma-Aldrich). The larvae were incubated in the dark for 1 h at 28 °C in 10 μg/ml AO solution in E3 and washed extensively before imaging.

#### GM1 ganglioside staining in zebrafish

To visualize GM1 gangliosides, a staining with Cholera Toxin subunit B (CtxB recombinant) conjugated with Alexa Fluor 549 or Alexa Fluor 647 (ThermoFisher Scientific) was performed on transplanted 4 dpf zebrafish embryos as previously reported (Zareba et al.).

#### LipidTOX

To visualize neutral lipids, HCS LipidTOX Deep Red (ThermoFisher Scientific) was added 1:1000 to live 4 dpf embryos for 1 h at 28 degrees. The embryos were rinsed and mounted for live imaging.

### RNA sequencing

#### Library preparation

Total RNA was extracted from mature iMGL (1*106 cells) using both the RNeasy Micro kit (Qiagen) or the Direct-zol RNA miniprep (Zymo Research), according to the manufacturers’ protocols. The quality of the isolated RNA was determined with a Qubit® (1.0) Fluorometer (Life Technologies, California, USA) and a Fragment Analyzer (Agilent, Santa Clara, California, USA). Only those samples with a 260 nm/280 nm ratio between 1.8 and 2.1 and a 28S/18S ratio within 1.5–2 were further processed. The TruSeq Stranded mRNA (Illumina, Inc, California, USA) was used in the succeeding steps. Briefly, total RNA samples (100–1000 ng) were polyA enriched and then reverse-transcribed into double-stranded cDNA. The cDNA samples was fragmented, end-repaired, and adenylated before ligation of TruSeq adapters containing unique dual indices (UDI) for multiplexing. Fragments containing TruSeq adapters on both ends were selectively enriched with PCR. The quality and quantity of the enriched libraries were validated using Qubit® (1.0) Fluorometer and the Fragment Analyzer (Agilent, Santa Clara, California, USA). The product is a smear with an average fragment size of approximately 260 bp. The libraries were normalized to 10 nM in Tris-Cl 10 mM, pH8.5 with 0.1% Tween 20.

#### Cluster generation and sequencing

The Novaseq 6000 (Illumina, Inc, California, USA) was used for cluster generation and sequencing according to standard protocol. Sequencing was single end 100 bp.

### Xenotransplantations of mature iMGL into Zebrafish embryos

Two days post fertilization (dpf), zebrafish embryos were anaesthetized and embedded ventrally (head up) as single embryos in small droplets of 1% low-melting agarose. The excess of agar was removed using a scalpel. Mature iMGL were harvested and filtered (mesh size 30–50 µm) after resuspension in HBSS with 1% Phenol Red to a final concentration of ~2.4*10^6^ cells/ml. The cell suspension was loaded into a 30 µm beveled needle and injected directly into the optic tectum of the mounted embryos using an oil injector (Eppendorf). 10–20 min after injection, the embryos were unmounted and used for further experiments.

### Light microscopy

Confocal, spinning disk (Andor Dragonfly 200 Sona spinning-disc microscope) or light sheet microscopy (Leica Viventis LS2 Live microscope, Bruker Luxendo TruLive3D Imager) were used to image the in vitro and in vivo samples, as specified below.

### Light microscopy of cells

Confocal analysis of live or fixed cells was performed with an Andor Dragonfly 200 Sona spinning-disc microscope with a Nikon 20×/NA 0.95 water or 40×/NA 1.25 silicon oil objectives and 40 μm spinning disc or a Leica SP8 with a 20×/NA0.75 or 40×/NA1.1 water objectives to capture stacks with a z-step of 0.5–1 μm. All images were analyzed in Fiji^61^ or Imaris (Oxford Instruments).

### Light microscopy of zebrafish embryos

Embryos were anaesthetized during mounting procedures and experiments using 0.01% tricaine (Merck) and pre-screened based on the expression of the desired fluorophore (when appropriate), using a Nikon ZMZ18 fluorescent stereoscope. Embryos were embedded in 1–1.5% low-melting (LM) agarose (PeqGOLD Low Melt Agarose, PeqLab Biotechnologie GmbH), dissolved in E3 medium with 0.01% tricaine and mounted as appropriate for the microscope used. For confocal microscopy, an Andor Dragonfly 200 Sona spinning-disc microscope with a Nikon 20x/NA 0.95 water or 40×/NA 1.25 silicon oil objectives and 40 μm spinning disc or a Leica SP8 with a 20×/NA0.75 or 40×/NA1.1 water objectives to capture stacks with a z-step of 0.5–1 μm. The same conditions were used for fixed zebrafish embryos. For light sheet microscopy, we used either a Leica Viventis LS2 Live microscope with two Nikon 10×/0.2 NA objectives or a Bruker Luxendo TruLive3D Imager with two Nikon CFI Plan Fluor 10×/0.3 NA illumination objectives and a Nikon CFI APO LWD 25×/1.1 NA detection objective, coupled with a tube lens (f = 400 mm) to acquire images at 50× magnification. For time lapse images, the stacks were taken at an interval of 30 s, 1 min, 3 min, or 5 min. All images were analyzed in Fiji (Schindelin, J. et al.) or Imaris.

### Chemical perturbations

#### U18666a

3dpf zebrafish embryos were incubated in 100 μM U18666a (Merck, Sigma-Aldrich) solution overnight at RT. Embryos were mounted and imaged.

#### Metronidazole

To induce apoptosis of endogenous zebrafish microglia, *slc37a2*^*NY007*^ TgBAC(fms:Gal4,UAS:nfsB-mCherry) 3 dpf embryos were incubated with 10 mM Metronidazole (MTZ, Merck, Sigma-Aldrich) solution in E3 medium with 0.2% (v/v) DMSO for 24 h. Embryos were washed extensively with E3 before mounting and imaging.

### Laser mediated injury

Injuries in the brain were performed at the Olympus IXplore SpinSR10 spinning disk confocal microscope equipped with the photomanipulation unit (Rapp OptoElectronic) using a 355 nm ablation laser (pulsed, UGA‑42 Caliburn). For imaging, a 60×/1.3 NA objective was used, and for injury, a circular pattern with 3% laser intensity was used.

### Electron microscopy (EM)

#### TEM on iMGL

Transmission Electron Microscopy on iMGL (control or fed with latex beads) was performed at the Center for Microscopy and Image Analysis (ZMB) at the University of Zurich. Cells were grown as by differentiation protocol in 6 well plates and fixed with 2.5% glutaraldehyde in 0.1 M sodium cacodylate buffer (pH 7.35, pre-warmed to 37 °C), centrifugated at 3000 rpm for 6 min and washed in cacodylate buffer followed by 1% OsO4 for 1 h in 0.1 M cacodylate buffer at 0 °C, and 1% aqueous uranyl acetate for 1 h at 4 °C. Afterwards, cells were then first embedded in 2% agar, dehydrated in an ethanol series, followed by propylene oxide, and embedded in Epon/Araldite (Sigma-Aldrich). Ultrathin (70 nm) sections were post-stained with lead citrate and examined with a Talos 120 transmission electron microscope at an acceleration voltage of 120 KV using a bottom mounted Ceta camera and the MAPS software for automatic image acquisition (Thermo Fisher Scientific, Eindhoven, The Netherlands).

#### Array tomography of iMGL

Serial ultrathin sections (100 nm) were collected on 10 mm ×20 mm silicon wafers using an ultramicrotome (Artos 3D, Leica Microsystems, Vienna, Austria)^62^ and contrasted with lead citrate for 7 min.

Sections were imaged in an Apreo 2 VS scanning electron microscope using the MAPS software package for automatic serial section recognition and image acquisition (Thermo Fisher, Eindhoven, The Netherlands). The array tomography workflow includes serial sections recognition, image region definition, autofunctions, and image acquisition^63^. Region of interest was imaged on every section using the OptiPlan mode of the system and the T1 detector with the following parameters: Pixel size of 4 nm, pixel dwell time of 1 us, electron high tension of 1.8 keV, beam current of 0.1 nA. Serial section Tiff images were aligned using the plugin TrakEM2^64^ in Fiji^61^.

### Electron microscopy on human iPSC-microglia in human brain organoids

#### Fixed sample processing

Microglia-containing forebrain organoids were fixed at 6/7 weeks post integration using 2% PFA/2% Glutaraldehyde in 0.1 M Phosphate Buffer (pH = 6.8) for 2 h at room temperature, followed by two washes in 0.1 M Phosphate Buffer (pH = 6.8). The fixed organoid samples were rinsed in 0.1 M cacodylate buffer, and the localization of fluorescently labeled cells was determined by widefield fluorescence microscopy for tdTomato. The sample was microdissected for the fluorescently labeled region and embedded in 4% low melting point agarose made with 1× PBS. A Leica VT1000 vibratome was used to collect 100 µm sections of the sample. Sections were stored in cryoprotectant (4:3:3—PBS:ethylene glycol:glycerol) at −20 °C until further processing. The sample was imaged on a Zeiss 880 microscope with a 20× air objective (NA 0.8) using the Airyscan detector. A comprehensive map of all tdTomato+ cells was generated using tile scanning and z-stack modules (reporting pixel size and volume size of whole ROI and sub-ROIs).

### vEM sample preparation

Materials were sourced from Electron Microscopy Sciences (Hatfield, PA) unless otherwise stated. Samples were rinsed repeatedly with ice-cold buffer (0.1 M sodium cacodylate, 3 mM calcium chloride) before further fixation with reduced osmium (1% osmium tetroxide, 1.5% potassium ferrocyanide, 0.1 M sodium cacodylate, 3 mM calcium chloride). Samples were rinsed repeatedly with ice-cold water and stained with 1% aqueous uranyl acetate for 1 h. Samples were rinsed thoroughly with ice cold water and serially dehydrated in ascending concentrations of ice-cold ethanol. Samples were finally rinsed in three changes of anhydrous ethanol at room temperature before infiltration with a 1:1 mixture of anhydrous ethanol and epoxy resin (Eponate 12, hard formulation; Ted Pella) for 4 h on a rotating mixer, followed by infiltration in pure resin overnight on a rotating mixer. The samples were embedded in two steps: first, the infiltrated sample was polymerized overnight at 70 °C on polypropylene bottlecap in a thin layer of resin with a gel capsule pressed into the resin around the sample. The following day, the gel capsule was backfilled with more resin and left to polymerize for another 48 h.

Serial ultrathin sections (100 nm) were collected from the blockface. Briefly, the blockface was trimmed using a 90° diamond trimming knife (Diatome) around the entire tissue section to a frustum of approximately 80 µm in height. A silicon chip (35 × 7 mm; University Wafer, Boston, MA) was hydrophilized in a plasma cleaner (Harrick), rinsed in pure water, and partially immersed in a Diatome Histo knife, with one end sticking out of the water at the back of the boat. Four drops of pure ethanol were added to the water in the boat to attenuate surface tension, and an ionizing gun (Leica EM Crion) was activated and oriented towards the cutting edge of the knife mounted on the ultramicrotome. Ribbons of approximately 100–150 serial sections of a nominal 100 nm thickness were collected onto a series of chips, for a total of approximately 650 sections or 65 µm of tissue volume. When ribbons of sufficient quality and length were generated, they were released from the knife edge using a single-eyelash brush and carefully positioned over the chip. The water level was then slowly lowered, and sections were allowed to dry down on the silicon substrate over a few minutes. Chips were further dried on a hot plate set to 60 °C for approximately 5 min.

### vEM imaging

Samples were loaded into a Zeiss Sigma VP scanning EM microscope (SEM) and imaged the array tomography software module of Atlas5 (FIBICS). Images were collected using a backscattered electron detector (Gatan) at a working distance of approximately 6 mm, with accelerating voltage set to 3 kV, a 30 µm aperture, and the beam in high current mode. Low magnification (50–100 nm/px) overviews of each section were collected and aligned using TrakEM2^64^ in Fiji^61^. These stacks were used for correlation with light microscopy, and subsequent selection of regions of interest for high resolution vEM. Correlative light-electron microscopy (CLEM) was achieved using BigWarp^65^ in Fiji^61^ between the tdTomato fluorescence and the microglia in the vEM. In sparse vEM regions, the only cells present were microglia, which had stereotypical ultrastructural features (e.g., electron dense ER, large clear endosomes, and complexes formed from lipid droplets). The presence of these distinguishing features supported the positive identification of tdTomato+ microglia in the vEM data from more dense cellular regions.

### EM segmentation and visualization

Regions of interest, including positively identified tdTomato+ microglia, were imaged at high resolution (8 nm/px) through continuous serial sections and aligned as described above. For segmentation of human cells in in vitro monoculture, first, EM image stacks were registered using the StackReg FIJI plugin^66^. For segmentation, we used the scikit-image toolkit^67^. For distinguishing between cytosol, background, and gastrosome, we thresholded based on the mean intensity of SLIC super-pixels^68^ (parameters: n_seg=20000, compactness_slic=0.2, sigma_slic=4, min_size_factor_slic=0.5, max_size_factor_slic=3). For segmenting the nucleus, we used a seeded watershed; the seeds were manually defined in napari^69^ and interpolated between layers. Rough masks were created in napari to exclude neighouring cells. The resulting segmentation was checked in napari and manually improved it where appropriate. For segmentations of human iPSC-derived microglia in brain organoids, because of the lower contrast of this data set, the slices of the EM stack were segmented manually in napari. After segmentation, in both cases the resulting masks were imported in Imaris (RRID:SCR_007370) for visualization purposes.

### Data analysis

#### RNA sequencing

In-house RNA-seq raw reads were processed using ARMOR (PMC6643886). Reads were aligned and counted against the human genome GRCh38 assembly, and Gencode release 43 annotation using salmon v1.4.0 and STAR 2.7.7a. Abud’s and iPSC data were downloaded from the Sequence Read Archive (SRA) accessions SRP092075 and SRP155574, respectively, using recount3 (PMID 34844637), with GRCh38 as the reference genome and Gencode’s annotation. The salmon-generated in-house count data were modeled with quasi-likelihood (QL) negative binomial generalized log-linear models, and differential expression analysis was performed using edgeR v3.36.0 on salmon outputs. Multi-dimensional scaling (MDS) plots were generated based on normalized count data using edgeR v3.36.0. Briefly, MDS plots were used to depict similarities between samples (including replicates and batches) in an unsupervised manner, showing the two leading fold-change dimensions which explain the largest proportion of variation in gene expression across samples. Batch correction was performed using ComBat from the R package sva v3.42.0, with the origin (Abud’s or in-house) used as the batch variable. Gene expression plots were generated to depict logCPMs as calculated by edgeR’s cpm(assay(x, ‘counts’), log = TRUE, prior.count = 2) or scaled and centered logCPMs, as reported in figure legends. Gene set enrichment analyses were performed using camera from limma v3.50.3 (PMC3458527) and mSigDB annotations on differentially expressed genes as reported by edgeR. Selected gene sets included: C2, curated gene sets from online pathway databases, publications in PubMed, and domain expert knowledge; C5, Gene Ontology; and C8, cell type signatures.

#### Transplants statistics

For each transplantation experiment, the total number of injected embryos was recorded. All embryos were imaged, and transplantation efficiency was calculated as the percentage of embryos showing zf-hiMG in the optic tectum (i.e., transplanted and correctly localized) over the total number injected. These embryos were then used to quantify the number of transplanted cells per embryo by manual counting in Fiji^61^. For survival analyses, embryos were repeatedly imaged from 2 days post-fertilization (dpf) to 5 dpf (corresponding to 0–3 days post-injection, dpi). Embryos were unmounted and remounted daily. Survival was expressed as the percentage of remaining cells over time, normalized to the initial number of cells at 0 dpi.

#### Morphology analysis

Morphological features of iMGL and zf-hiMG were manually analyzed on 2D maximum intensity projections using Fiji^61^. The area of the cell body was measured using the segmented line tool. To estimate the total area covered by a cell, the convex hull was manually drawn by connecting the distal tips of the branches to form the smallest possible polygon encompassing the entire cell. Additionally, the number of primary branches directly emerging from the cell body was manually counted.

#### Branch motility

Branch motility was quantified on 2D maximum intensity projections from light-sheet microscopy data. When cells were migrating, the imaging frame was adaptively cropped to keep the cell centered. Raw 3D volumes were converted to H5 format using Fiji BigDataProcessor^70^. Individual branches were tracked manually using the mTrackJ plugin^71^ in Fiji^61^, both in vitro and in vivo.

#### Cell body motility

Cell body motility was quantified in 2D using using the mTrackJ plugin^71^ in Fiji^61^. For the qualitative representation of zf-hiMG cell body motility, microglia were tracked semi-automatically using Imaris spot detection over time. Spot detection was automated and manually corrected to ensure accurate placement and tracking throughout the time-lapse.

#### Acridine Orange (AO) quantification

Quantification of uncollected apoptotic neurons was performed using acridine orange (AO) staining (described above) and Imaris spot detection. 3D volumes were analyzed to detect AO⁺ spots within the optic tectum, using a spot diameter of 4 μm. Automated detection was manually corrected. In transplanted samples, the segmentation channel was duplicated and overlaid with the zf-hiMG channel to exclude AO⁺ spots located within zf-hiMG. In experiments with green zf-hiMG, AO⁺ clusters recognizable as single, intense aggregates were excluded from quantification, as they were considered internalized by microglia. This exclusion procedure was applied when needed to both the WT and mutant groups. In non-transplanted samples, the segmentation channel was overlaid with the endogenous microglia channel to exclude AO^+^ spots located within microglia.

#### Fluorescence intensity

To confirm colocalization of apoptotic material within vesicular compartments (e.g., phagosomes, gastrosomes), fluorescence intensity profiles were extracted in Fiji^61^. A line was drawn across the vesicle of interest, and intensity values for both the zf-hiMG signal and the apoptotic neuron signal were recorded and overlayed.

#### Gastrosome diameter

The diameter of gastrosomes was quantified manually in Fiji^61^ on 3D image stacks. For each cell, the entire volume was scanned, and the largest vesicle was identified. Using the straight line tool, the longest possible axis within the vesicle was measured and recorded as the gastrosome diameter. Only the largest vesicle per cell was included in the analysis.

#### Statistical analysis and reproducibility

Statistical analyses were performed using Prism 9 (GraphPad), Python, or R. Unless otherwise specified, conditions were compared using an unpaired, two-tailed, nonparametric Mann-Whitney U test with Bonferroni correction for multiple comparisons. To report statistical significance, *p*-value thresholds listed in Table S5 were used. In all boxplots, the whiskers indicate the minimum and maximum values. Information on sample size (indicated as n) and number of replicates (indicated as N) can be found in figure legends.

### Reporting summary

Further information on research design is available in the Nature Portfolio Reporting Summary linked to this article.

## Supplementary information

Supplementary information

Description of Additional Supplementary Materials

Supplementary Data 1

Video 1

Video 2

Video 3

Video 4

Video 5

Video 6

Video 7

Video 8

Video 9

Video 10

Video 11

Reporting Summary

## Data Availability

Numerical data related to this manuscript are provided in Supplementary Data 1. The imaging data generated in this study are available from the corresponding author on reasonable request. Raw and processed RNA-seq data generated in this study are available at GEO GSE324751. Reanalyzed data were downloaded from SRA accessions SRP092075 and SRP155574. Code to analyze the RNA-seq data is available at https://zenodo.org/records/18997850 under the GPLv3 terms.
