# Supplementary material for: A scalable human-zebrafish xenotransplantation model reveals gastrosome-mediated processing of dying neurons by human microglia
Source: Commun Biol. 2026 Apr 9;9:785. doi: 10.1038/s42003-026-09948-6 (PMC13250125; doi:10.1038/s42003-026-09948-6)
Supplement: Supplementary file 1 — Supplementary information [file 42003_2026_9948_MOESM1_ESM.pdf]

Supplementary Information

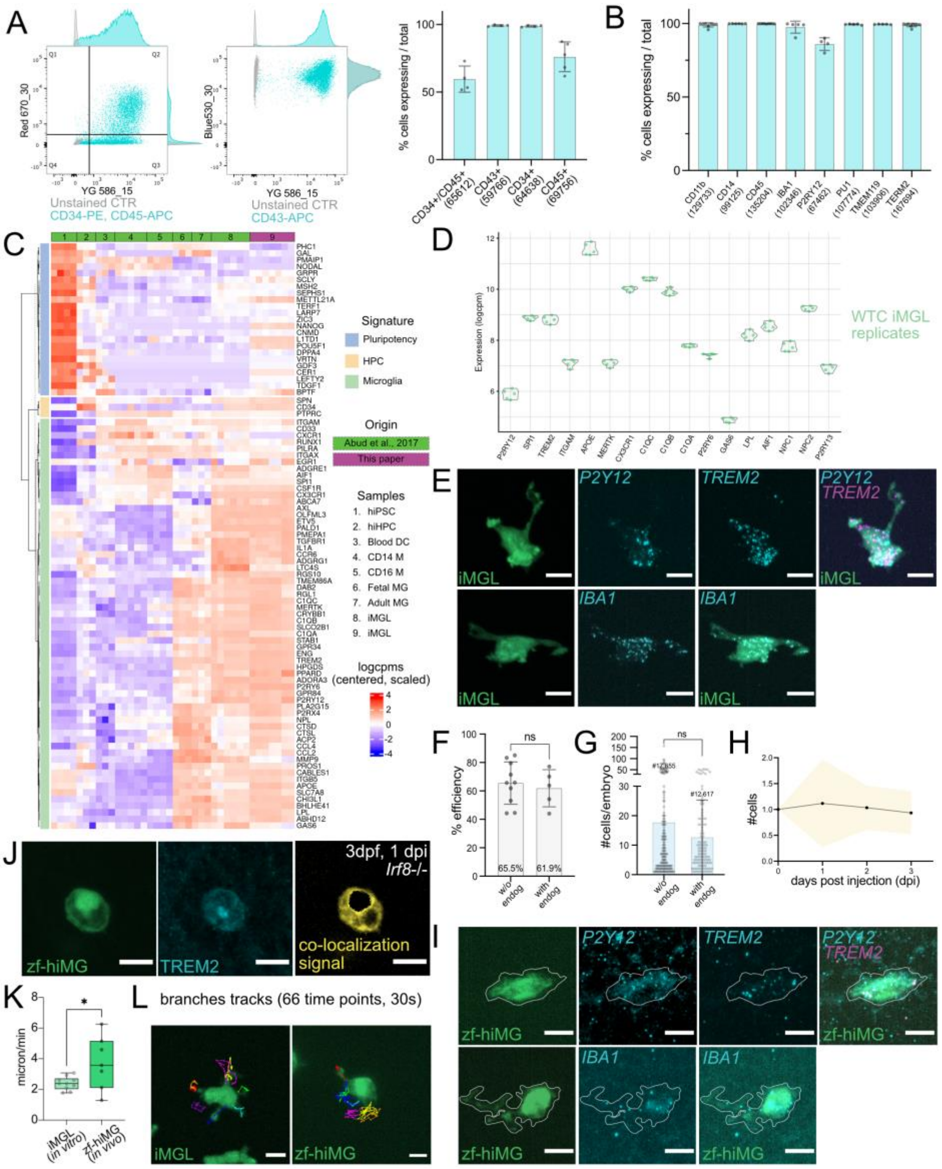

Supplementary Figure 1. Characterization and validation of iMGL and their engraftment in zebrafish (zf-hiMG)
(A) Flow cytometry analysis of iHPC generated from fluorescently labelled hiPSC, based on typical HPC marker expression; N=5 (experiments), number of cells in x axes. (B) Histogram showing FACS quantification of microglial protein markers in iMGL; N=9 (experiments), number of cells in x axes. (C) Heatmap of bulk RNA-seq data comparing WT iMGL generated in this study to published datasets of iMGL and other cell types (Abud et al. 2019[1]). (D) Violin plots showing normalized expression levels of canonical microglial genes in WT iMGL. (E) In vitro Hybridization Chain Reaction (HCR) of key microglial transcripts in WT iMGL; scale bars 10 µm. (F–G) Quantification of xenotransplantation efficiency. (F) Percentage of Irf8st95 embryos containing fluorescently labelled iMGL in the Optic Tectum (OT); N=10, n=530 (w/o endogenous); N=5, n=190 (with endogenous), p = 0.5941, unpaired two-tailed Mann-Whitney test. (G) Number of zf-hiMG per embryo; N=8, n=171 (w/o endogenous); N=5, n=107 (with endogenous), p = 0.9596, unpaired two-tailed Mann-Whitney test. (H) Quantification of zf-hiMG every 24hrs, from 2 dpf, 0 dpi to 5 dpf, 3 dpi; N=2, n=56. (I) In vivo HCR on zf-hiMG of human specific microglial transcripts TMEM119, P2Y12, IBA1, and TREM2; scale bars 10 µm. (J) In vivo immunofluorescence staining of human TREM2 in zf-hiMG; scale bar 10 µm. (K–L) Analysis of zf-hiMG motility (in vivo) compared to iMGL (in vitro). (K) Quantification of branch extension/retraction speed in vitro versus in vivo; N=3, n=10 (in vitro), n=7 (in vivo), p = 0.0309, unpaired two-tailed t-test. (L) Qualitative tracking of branch motility over time in representative iMGL (left) and zf-hiMG (right); scale bars 10 µm. N=experiments, n=embryos (F-H)/cells (K). Microscopy data acquired using Andor Dragonfly 200 Sona spinning-disc microscope.

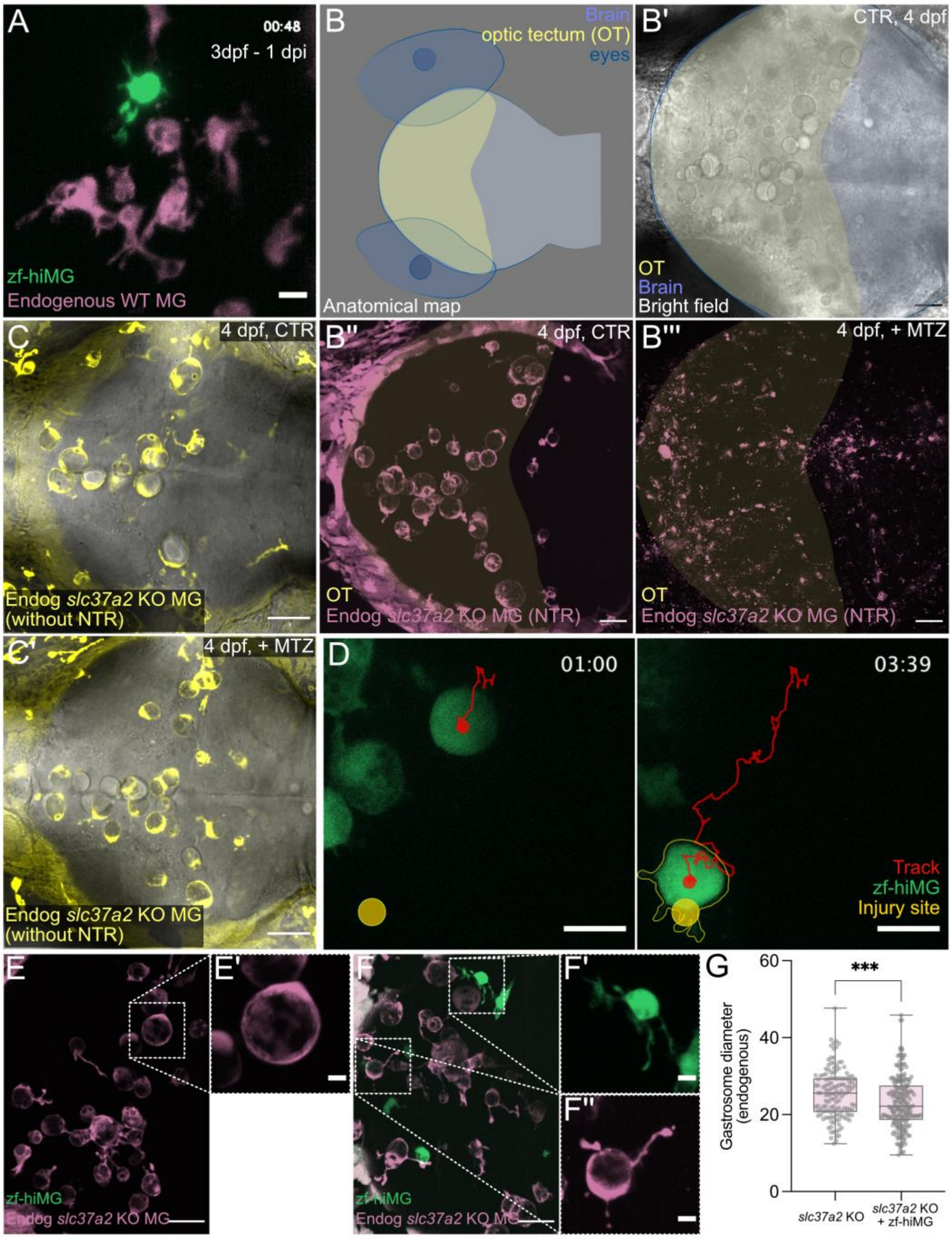

Supplementary Figure 2. Coexistence and functional complementation of human and zebrafish microglia in vivo. (A) GFP labelled WT zf-hiMG transplanted into WT zebrafish embryos with red-labelled endogenous microglia (Tg(fms:Gal4;UAS:nfsB-mCherry)), OT dorsal view, 3 dpf, 1 dpi; 3 minutes time resolution; scale bar 10 µm. (B) Dorsal view anatomical map of a zebrafish embryo showing segmented regions of the eyes, brain, and optic tectum (OT); (B') Bright field image of a control slc37a2 KO embryo at 4 dpf; (B'') Image of control slc37a2 KO endogenous microglia (Tg(fms:Gal4;UAS:nfsB-mCherry)) at 4 dpf; (B''') Image of MTZ-treated slc37a2 KO endogenous microglia (Tg(fms:Gal4;UAS:nfsB-mCherry)) at 4 dpf; scale bars 50 µm. (C–C') Fluorescence and bright field images of slc37a2 KO embryos at 4 dpf lacking NTR expression (Tg(mpeg1:eGFP-caax)) without (C) or with (C') MTZ treatment; scale bar 50 µm. (D) Injury response of GFP-labelled WT zf-hiMG in Irf8st95 4 dpf, 2 dpi zebrafish embryo; 5 minutes time resolution; scale bar10 µm. (E–F) Dorsal views of slc37a2 KO 4 dpf embryos (Tg(fms:Gal4;UAS:nfsB-mCherry)); scale bars 50 µm (overviews) and 10 µm (cropped zooms): (E) without zf-hiMG transplant; (F) with zf-hiMG transplant (2 dpi). (G) Quantification of E-F: gastrosome diameter of endogenous slc37a2 KO microglia ± zf-hiMG transplantation; N=7, n=138 (slc37a2 KO); N=9, n=202 (slc37a2 KO+zf-hiMG); N=embryos, n=cells, p = 0.0009, two-tailed Mann-Whitney test. Microscopy data acquired using Bruker Luxendo TruLive3D Imager and Andor Dragonfly 200 Sona spinning-disc microscope.

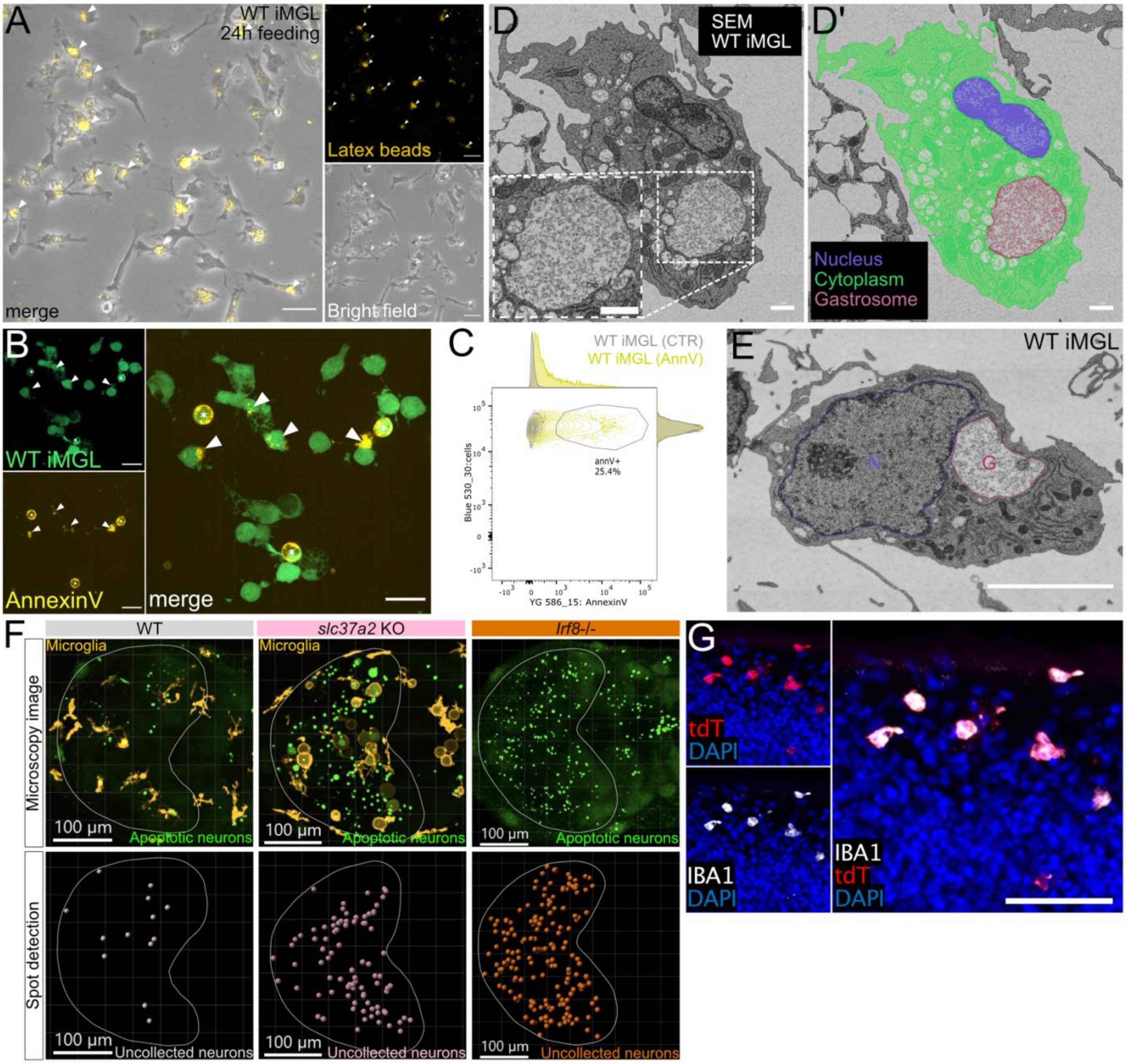

Supplementary Figure 3. In iMGL the gastrosome is a collective phagocytic compartment characterized by distinct ultrastructure characteristics.

(A) WT iMGL monoculture fed with 1 µm fluorescent latex beads and imaged after 24 h; scale bar 30 µm. (B) Light microscopy of WT iMGL monocultures stained with Annexin V to label apoptotic structures. Asterisks (*) indicate dead iMGL, while arrowheads mark Annexin V-positive debris within healthy, neighbouring iMGL; Scale bar: 20 µm. (C) Flow cytometry analysis of WT iMGL stained with Annexin V (yellow) compared to unstained control (gray). (D) Singe slice from an array SEM of WT iMGL in vitro (monoculture) with segmentation (D') featuring the cytoplasm (green), nucleus (blue) and gastrosome (purple); scale bars 1 µm. (E) Electron micrograph single plane of WT iMGL in monoculture; N = nucleus, G = gastrosome; scale bar 5 µm. (F) Representative images (quantified in Fig. 3E') of uncollected AO+ apoptotic nuclei in the optic tectum of wt, slc37a2 and Irf8st95 mutant embryos. Microglia are in orange (mpeg:Gal4; UAS:lyntag-RFPt). Lower panels have been obtained with Imaris spot detection, using a spot diameter of 4 µm; scale bar 100 µm. (G) IHF showing expression of IBA1 by tdT-positive (EMP-derived) organoid-resident microglia-like cells. DAPI has been used to stain nuclei. ; scale bar 50 µm. Microscopy data acquired using Andor Dragonfly 200 Sona spinning-disc microscope (B and F) and Leica THUNDER microscope (G).

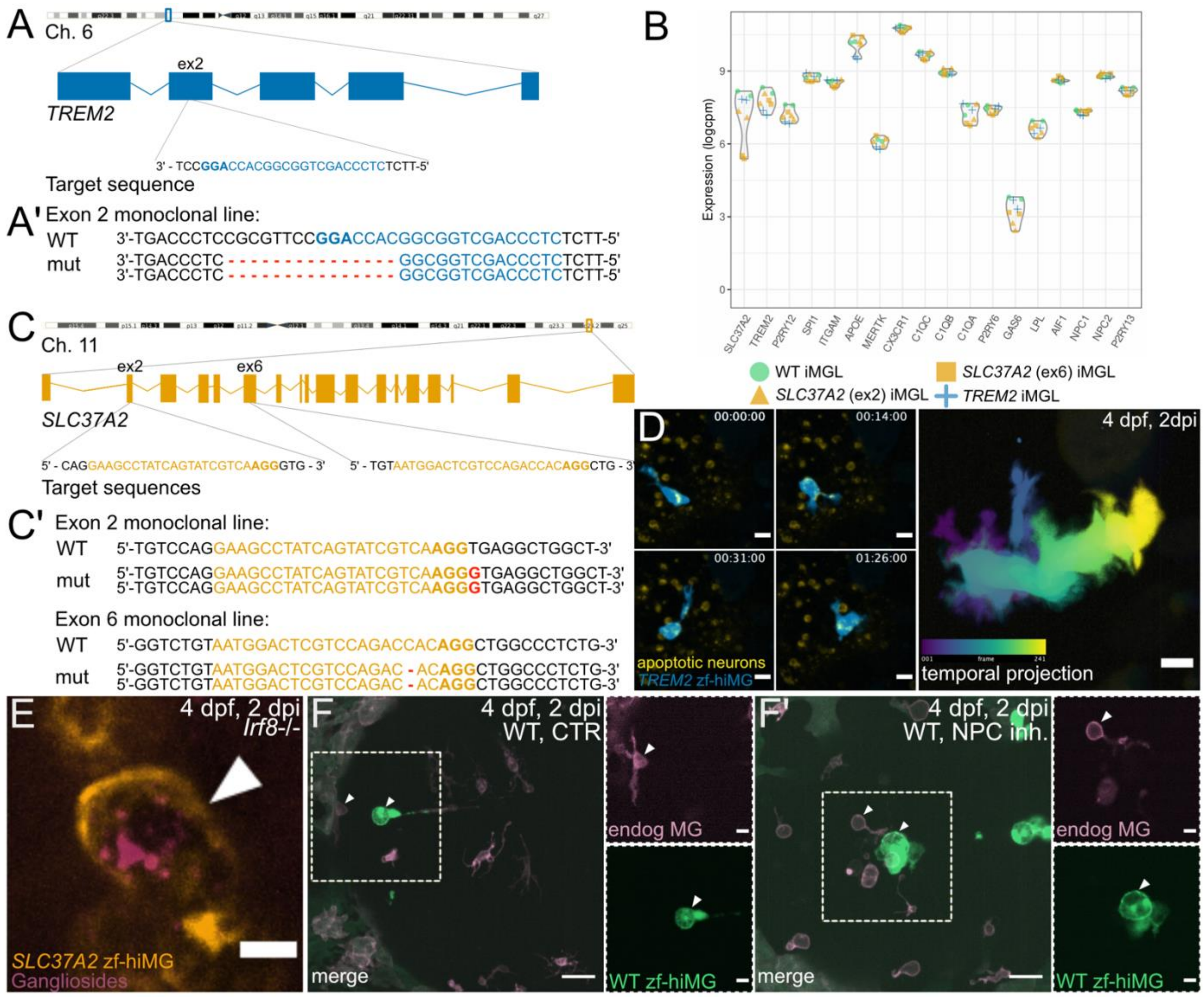

Supplementary Figure 4. In vitro and in vivo characterization of perturbed human microglia-like cells.

(A) Schematic of CRISPR-Cas9–mediated targeting strategy for TREM2 in GFP labelled human iPSCs, using a guide RNA designed to exon 2. (A’) Sequencing results of the TREM2 mutant clone (targeted exon). (B) Bulk RNA-sequencing analysis of WT, SLC37A2 mutant and TREM2 mutant iMGL, showing reduced SL37A2 transcript levels in SLC37A2 mutants compared to WT (ex2 $\log_2$FC = –1.63, p = 0.000093, FDR = 0.056; not significant after correction; ex6 $\log_2$FC = –3.37, p = 0.000000162, FDR = 0.00030, significant after correction), reduced TREM2 transcript levels in TREM2 mutants compared to WT ($\log_2$FC =–1.37, p = 0.0000343, FDR = 0.0147 , significant after correction) and preserved expression of canonical microglial markers for all genotypes. (C) Schematic of CRISPR-Cas9 targeting strategy for SLC37A2 in GFP-labelled human iPSC, using a guide RNA designed to exon 2 and exon 6. (C’) Sequencing results of the two SLC37A2 mutant clones (targeted exons). (D) Time lapse images of GFP-labelled TREM2 zf-hiMG in Irf8st95 embryos with neuronal apoptotic marker (Tg(nbt:dLexPRLexOP:secA5-BFP); temporal projection of the same cell (right panel); dorsal view; 30 seconds time resolution; 4dpf, 2 dpi; scale bars 10 µm. (E) CtxB staining to detect GM1 gangliosides in GFP-labelled SLC37A2 zf-hiMG xenotransplanted into Irf8st95 zebrafish embryos; 4 dpf, 2 dpi; dorsal view; scale bar 10 µm. (F-F') Morphological comparison of NPC1 inhibition (F’) versus control (F) in GFP-labelled WT zf-hiMG transplanted into WT embryos with microglia labelled with Tg(fms:Gal4;UAS:lyn-miRFP670); 4 dpf, 2 dpi; dorsal view; scale bars scale bars 30 µm (overview) and 10 µm (zoomed crop). Microscopy data acquired using Andor Dragonfly 200 Sona spinning-disc microscope (A, E, E’, F).

Gene	Targeted Exon	crRNA (5'-3')	PAM	Transcript ID	Reference
TREM2	Exon2	CTCTCCCAGCTGGCGGCACC	AGG	ENST00000373113.8	Mc Quade et al., 2020[2]
Slc37a2	Exon2	CTATCAGTATCGTCAAGGTG	AGG	ENST00000403796.7	NA
Slc37a2	Exon6	AATGGACTCGTCCAGACCAC	AGG	ENST00000403796.7	NA

Table S1. List of CRISPR sequences

Target	Primer	Sequence (5'-3')	Tm (°C)
TREM2	forward	CTGCAGAGCAAGCAAGAGTG	52
	reverse	CTGGTAGAGACCCGCATCAT	
SLC37A2 (exon 2)	forward	AGAGTTGGTGGTCCTCAGGC	54
	reverse	AAAGCAGCCACATCAGACCC	
SLC37A2 (exon 6)	forward	ACTTCCAATGCACGAGAGGC	54
	reverse	CCTGCTCTATGGGTTAAAGCC	

Table S2. List of primers for CRISPR sequencing

Gene of interest	NCBI accession number	HCR Amplifier	Hairpins
P2RY12	NM_022788.5	B2	red (λ=594nm),<br>far red (λ=647nm)
AIF1	NM_001623.5	B1	red (λ=594nm),<br>far red (λ=647nm)
TREM2	NM_018965.4	B1	red (λ=594nm),<br>far red (λ=647nm)

Table S3. List of genes of interest, amplifiers and hairpins for HCR experiments

Gene	NCBI Accession Number	Sequence
TREM2	NM_018965.4	gatcctctcttttctgcagttcaagggaaagacgagatcttgcacaaggcACTCTGCTTCTGCCCTTGGCTGGGGAAGGGTGGCATGGAGCCTCT<br>CCGGCTGCTCATCTTACTCTTTGTCACAGAGCTGTCCGGAGCCCACAACACCACAGTGTTCCAGGGCGTGGCGGCCAGTCCCT<br>GCAGGTGTCTTGCCCCTATGACTCCATGAAGCACTGGGGGAGGCGCAAGGCCTGGTGCCGCCAGCTGGGAGAGAAGGGCCC<br>ATGCCAGCGTGTGGTCAGCACGCACAACTTGTGGCTGCTGTCCTTCCTGAGGAGGTGGAATGGGAGCACAGCCATCACAGAC<br>GATACCCTGGGTGGCACTCTCACCATTACGCTGCGGAATCTACAACCCCATGATGCGGGTCTCTACCAGTGCCAGAGCCTCCAT<br>GGCAGTGAGGCTGACACCCTCAGGAAGGTCCTGGTGGAGGTGCTGGCAGACCCCCTGGATCACCGGGATGCTGGAGATCTCT<br>GGTTCCCCGGGGAGTCTGAGAGCTTCGAGGATGCCCATGTGGAGCACAGCATCTCCAGGAGCCTCTTGGAAGGAGAAATCCC<br>CTTCCCACCCACTTCCATCCTTCTCCTCCTGGCCTGCATCTTTCTCATCAAGATTCTAGCAGCCAGCGCCCTCTGGGCTGCAGCCT<br>GGCATGGACAGAAGCCAGGGACACATCCACCCAGTGAACTGGACTGTGGCCATGACCCAGGGTATCAGCTCCAAACTCTGCC<br>AGGGCTGAGAGACACGTGAAGGAAGATGATGGGAGGAAAAGCCCAGGAGAAGTCCCACCAGGGACCAGCCCAGCCTGCATA<br>CTTGCCACTTGGCCACCAGGACTCCTTGTTCTGCTCTGGCAAGAGACTACTCTGCCTGAACACTGCTTCTCCTGGACCCTGGAAG<br>CAGGGACTGGTTGAGGGAGTGGGGAGGTGGTAAGAACACCTGACAACTTCTGAATATTGGACATTTTAAACACTTACAAATAA<br>ATCCAAGACTGTCATATTTAGCTGGAtagttttgggcatcatgaaagtctcattttcttccacaggggtaagaaaa
P2RY12	NM_022788.5	acacaacaaacatcatagtgctgaaatgacaaaaatccagggtagtgatcACAATCAGAAGACAGGAGCTGCAGAACAGAACACTTTCTCATGT<br>CCAGGGTCAGATTACAAGAGCACTCAAGACTTTACTGACGAAAACTCAGGAAATCCTCTATCACAAAGAGGTTTGGCAACTAA<br>ACTAAGACATTAAAAGGAAAATACCAGATGCCACTCTGCAGGTTGCAATAACTACTACTTACTGGATACATTCAAACCCTCCAG<br>AATCAACAGTTATCAGGTAACCAACAAGAAATGCAAGCCGTCGACAACCTCACCTCTGCGCCTGGTAACACCAGTCTGTGCACC<br>AGAGACTACAAAATCACCCAGGTCCTCTTCCCACTGCTCTACACTGTCCTGTTTTTTGTTGGACTTATCACAAATGGCCTGGCGA<br>TGAGGATTTTCTTTCAAATCCGGAGTAAATCAAACTTTATTATTTTTCTTAAGAACACAGTCATTTCTGATCTTCTCATGATTCTG<br>ACTTTTCCATTCAAAATTCTTAGTGATGCCAAACTGGGAACAGGACCACTGAGAACTTTTGTGTGTCAAGTTACCTCCGTCATAT<br>TTTATTTCACAATGTATATCAGTATTTCATTCCTGGGACTGATAACTATCGATCGCTACCAGAAGACCACCAGGCCATTTAAAAC<br>ATCCAACCCCAAAAATCTCTTGGGGGCTAAGATTCTCTCTGTTGTCATCTGGGCATTCATGTTCTTACTCTCTTTGCCTAACATGA<br>TTCTGACCAACAGGCAGCCGAGAGACAAGAATGTGAAGAAATGCTCTTTCCTTAAATCAGAGTTCGGTCTAGTCTGGCATGAA<br>ATAGTAAATTACATCTGTCAAGTCATTTTCTGGATTAATTTCTTAATTGTTATTGTATGTTATACACTCATTACAAAAGAACTGTA<br>CCGGTCATACGTAAGAACGAGGGGTGTAGGTAAAGTCCCCAGGAAAAAGGTGAACGTCAAAGTTTTCATTATCATTGCTGTAT<br>TCTTTATTTGTTTTGTTCCTTTCCATTTTGCCCGAATTCCTTACACCCTGAGCCAAACCCGGGATGTCTTTGACTGCACTGCTGAA<br>AATACTCTGTTCTATGTGAAAGAGAGCACTCTGTGGTTAACTTCCTTAAATGCATGCCTGGATCCGTTCATCTATTTTTTCCTTTG<br>CAAGTCCTTCAGAAATTCCTTGATAAGTATGCTGAAGTGCCCCAATTCTGCAACATCTCTGTCCCAGGACAATAGGAAAAAAGA<br>ACAGGATGGTGGTGACCCAAATGAAGAGACTCCAATGTAAACAAATTAACTAAGGAAATATTTCAATCTCTTTGTGTTCAGAAC<br>TCGTTAAAGCAAAGCGCTAAGTAAAAATATTAACTGACGAAGAAGCAACTAAGTTAATAATAATGACTCTAAAGAAACAGAAG<br>ATTACAAAAGCAATTTTCATTTACCTTTCCAGTATGAAAAGCTATCTTAAAATATAGAAAACTAATCTAAACTGTAGCTGTATTA<br>GCAGCAAAACAAACGACATCCAATTGTCATGCTGCATGCAAAACTACACAGAATTCATGTTTTGCAGAGTTTTGCCAAAATGAG<br>TAATCATATAATATTTACTGTAATTTTTAAAATACATTATCGTTCACAATTTTATTTTTTCATAATCAACTAAGGAAGAACGATCA<br>ATTGGATATAATCTTCTTACCAAAAATGATAGTTAAAATGTATATATATCCTAGTCCCCTAACCAAATCCTGACCTATTGGGATA<br>CTTATAAAAATTTAAGTAAGTGGGATACACAAAGAATAATAACTATTAACTTTTCATTATTAGCAAAAACCTAAGGGATTTAAAC<br>TAATTGAAACTGTATTTGATTGGACTTAATTTTTTATGTTTATTTAGAAGATAAAGATTTAAAGAAGACCTTTACAATAAAGAGA<br>AGAAATATCGAAGTCATTAAAATAAGGAGACTTACTTTTATGACATTCTAATACTAAAAAATATAGAAATATTTCCTTAATTCTA<br>GAGAAACTAGTTTTACTAATTTTTTACAACTTCAATAATACCATCATTGACACTTACCTTTATTAATTAGCTTCTAGAAAATAGCT

		GCTAATTAGGTTAATGAACATTTTACCTTAGTGAAAAAAATTAATTAAATATGATTACAAAGTTGCACAGCATAACTACTGAGAGGAAAGTGATTGATCTGTTTGTAATTACTTGTTTGTATTGGTGTGTATAAAATACAAAATTTACATTAAACTCTAAATCATTctttgttgtaaattctttgttcaaagatattagataccatgactcaccat
AIF1 (IBA1)	NM_001623.5	ctgggaggcagaggaggaaggaatgaggggaaaggggaagtttgggaggaAGGCTTCTGAGAAGACTGGTGGGAGAGAAGGAGAGCCTGCAGACAGAGGCCTCCAGCTTGGTCTGTCTCCCCACCTCTACCAGCATCTGCTGAGCTATGAGCCAAACCAGGGATTTACAGGGAGGAAAAGCTTTCGGACTGCTGAAGGCCCAGCAGGAAGAGAGGCTGGATGAGATCAACAAGCAATTCCTAGACGATCCCAAATATAGCAGTGATGAGGATCTGCCCTCCAAACTGGAAGGCTTCAAAGAGAAATACATGGAGTTTGACCTTAATGGAAATGGCGATATTGATATCATGTCCCTGAAACGAATGCTGGAGAAACTTGGAGTCCCCAAGACTCACCTAGAGCTAAAGAAATTAATTGGAGAGGTGTCCAGTGGCTCCGGGGAGACGTTCAGCTACCCTGACTTTCTCAGGATGATGCTGGGCAAGAGATCTGCCATCCTAAAAATGATCCTGATGTATGAGGAAAAAGCGAGAGAAAAGGAAAAGCCAACAGGCCCCCCAGCCAAGAAAGCTATCTCTGAGTTGCCCTGATTTGAAGGGAAAAGGGATGATGGGATTGAAGGGGCTTCTAATGACCCAGATATGGAAACAGAAGACAAAATTGTAAGCCAGAGTCAACAAATTAAATAAATTACCCCCTCCTCCAgatcaagtcagcttagtttttatttgggtgattttttcctgggtttggg

Table S4. List of HCR sequences

**p-value**	**Wording**	**Summary**
< 0.0001	Very highly significant	****
0.0001 – 0.001	Highly significant	***
0.001 – 0.01	Very significant	**
0.01 – 0.05	Significant	*
≥ 0.05	Not significant	ns

Table S5. Summary of Statistical Significance Thresholds and Reporting Symbols

Reagent type (species) or resource	Designation	Source or reference	Identifiers	Additional information
Genetic reagent (*Danio rerio*)	*irf8^{st95}*	Shiau, C. E. et al, 2015[3]	ZDB-FISH-150901-4256	
Genetic reagent (*Danio rerio*)	*slc37a2^{NY007}*	Villani et al, 2019[4]	ZDB-FISH-191226-5	
Genetic reagent (*Danio rerio*)	Tg(mpeg1:GFP-caax)	Villani et al., 2019[4]	ZDB-TGCONSTRCT-191211-1	
Genetic reagent (*Danio rerio*)	Tg(nbt:dLexPR-LexOP:secA5-BFP)	Mazaheri et al., 2014[5]	ZDB-TGCONSTRCT-170110-1	
Genetic reagent (*Danio rerio*)	TgBAC(fms:Gal4,UAS:nfsB-mCherry)	Gray et al., 2011[6]	ZDB-ALT-110707-2	
Genetic reagent (*Danio rerio*)	Tg(UAS:lyn-miRFP670)	This paper	NA	
Cell line	TC-mEGFP-Safeharborlocus(AAVS1)-cl6(mono-allelic tag)	Allen Institute for Cell Science	AICS-0036-006	
Cell line	WTC-mTagRFPT-CAAX-Safeharborlocus(AAVS1)-cl91 (mono-allelic tag)	Allen Institute for Cell Science	AICS-0054-091	
Commercial assay or kit	MultiSite Gateway Pro	ThermoFisher Scientific	12537100	
Commercial assay or kit	STEMdiff Hematopoietic Kit	STEMCELL Technologies	05310	
Commercial assay or kit	STEMdiff Microglia Differentiation Kit	STEMCELL Technologies	100-0019	
Commercial assay or kit	STEMdiff Microglia Maturation Kit	STEMCELL Technologies	100-0020	
Commercial assay or kit	Annexin V			
Commercial assay or kit	RNeasy Micro kit	Qiagen	74004	
Commercial assay or kit	Direct-zol RNA miniprep	Zymo Research	R2050	
Reagent	mTeSR1	STEMCELL Technologies	85850	
Reagent	MTeSR Plus	STEMCELL Technologies	100-0276	
Reagent	ReLeSR	STEMCELL Technologies	05872	
Reagent	Matrigel hESC-Qualified Matrix	Corning	354277	
Reagent	Matrigel Growth Factor Reduced (GFR) Basement Membrane Matrix	Corning	354230	
Reagent	Y-27632 (Rock-Inhibitor)	STEMCELL Technologies	05888	
Reagent	Accutase	STEMCELL Technologies	07922	
Reagent	Alt-R® CRISPR-Cas9 crRNA	IDT		
Reagent	Alt-R® CRISPR-Cas9 tracrRNA	IDT	IDT (1072533)	
Reagent	Alt-R® S.p. HiFi Cas9 Nuclease V3 (stock 61uM)	IDT	IDT (1081060)	
Reagent	Nuclease-free IDTE	IDT	IDT (11-01-01-01)	

Reagent	StemFlexTM Medium	ThermoFisher Scientific	A3349401	
Reagent	Human Stem Cell NucleofectorTM Kit 1	Lonza	VPH-5012	
Reagent	Alt-R® Cas9 Electroporation Enhancer	IDT	1075915	
Reagent	CloneR	STEMCELL Technologies	05888	
Reagent	TrypLE Express Enzyme (1X), no phenol red	ThermoFisher Scientific	12604013	
Reagent	Acridine Orange	Merck (Sigma-Aldrich)	A6014	
Reagent	CtxB 549	ThermoFisher Scientific	C22842	
Reagent	CtxB 647	ThermoFisher Scientific	C34778	
Reagent	HCS LipidTOX Deep Red	ThermoFisher Scientific	H34477	
Reagent	Tricaine	Merck	A5040	
Reagent	U18666a	Merck (Sigma-Aldrich)	U3633	
Antibody	Anti-human CD43	STEMCELL Technologies	60085AZ	1:100
Antibody	Anti-human CD45	STEMCELL Technologies	60018AZ.1	1:100
Antibody	Anti-human CD34	ThermoFisher Scientific	CD34-581-04	1:100
Antibody	Anti-human CD45	STEMCELL Technologies	60018AZ.1	1:200
Antibody	Anti-human CD11b	STEMCELL Technologies	60040PE.1	1:100
Antibody	Anti-human CD14	STEMCELL Technologies	60004AZ.1	1:200
Antibody	Anti-human TREM2	R&D Systems	AF1828	1:20 (FACS), 10µg/ml (IHC)
Antibody	Anti-human pU.1	Cell Signaling	2266S	1:40 (FACS), 1:1000 (IHC)
Antibody	Anti-human P2Y12	Merck	HPA014518	1:25
Antibody	Anti-human IBA1	Synaptic Systems	234003	1:250 (FACS), 1:1000 (IHC)
Antibody	Anti-goat AF546	ThermoFisher Scientific	A-11056	1:500
Antibody	Anti-rabbit AF568	Molecular Probes	A11011	1:500
Instrument	NucleofectorTM 2b	Lonza	AAB-1001	
Instrument	IsoCell-isoHub	IotaSciences		
Instrument	BD LSR II Fortessa Analyzer			
Instrument	CellTram 4r Oil	Eppendorf		
Instrument	Andor Dragonfly 200	Andor		
Instrument	SP8	Leica		
Instrument	Viventis LS2 Live	Leica		
Instrument	TruLive3D Imager	Brucker, luxendo		
Instrument	IXplore SpinSR10	Olympus		
Software	CHOPCHOP		Labun, K. et al, 2019	
Software	FlowJo			
Software	Fiji			
Software	Imaris	Oxford Instruments		

Table S6. Resource Table

Supprementary References

1. Abud, E. M. *et al.* iPSC-Derived Human Microglia-like Cells to Study Neurological Diseases. *Neuron* **94**, 278-293.e9 (2017).

2. McQuade, A. *et al.* Gene expression and functional deficits underlie TREM2-knockout microglia responses in human models of Alzheimer's disease. *Nat. Commun.* **11**, 5370 (2020).

3. Shiau, C. E., Kaufman, Z., Meireles, A. M. & Talbot, W. S. Differential Requirement for irf8 in Formation of Embryonic and Adult Macrophages in Zebrafish. *PLOS ONE* **10**, e0117513–e0117513 (2015).

4. Villani, A. *et al.* Clearance by Microglia Depends on Packaging of Phagosomes into a Unique Cellular Compartment. *Dev. Cell* **49**, 77-88.e7 (2019).

5. Mazaheri, F. *et al.* Distinct roles for BAI1 and TIM-4 in the engulfment of dying neurons by microglia. *Nat. Commun. 2014* **5**, 1–11 (2014).

6. Gray, C. *et al.* Simultaneous intravital imaging of macrophage and neutrophil behaviour during inflammation using a novel transgenic zebrafish. *Thromb. Haemost.* **105**, 811–819 (2011).
