# Supplementary material for: A scalable human-zebrafish xenotransplantation model reveals gastrosome-mediated processing of dying neurons by human microglia
Source: Commun Biol. 2026 Apr 9;9:785. doi: 10.1038/s42003-026-09948-6 (PMC13250125; doi:10.1038/s42003-026-09948-6)
Supplement: Supplementary file 2 — Description of Additional Supplementary Materials [file 42003_2026_9948_MOESM2_ESM.docx]

**Description of Additional Supplementary Files**

**File name:** Supplementary Data 1

**Description:** Numerical data related to the following measurements: branches per zf-iMG cell, branch length, convex hull area - cell body, branches area, convex hull area - whole cell, Uncollected apoptotic neurons in Irf8-/- per fish with and without zf-hiMG, Gastrosome diameter of zf-hiMG in different genetic backgrounds, uncollected apoptotic neurons per fish in different backgrounds, uncollected apoptotic neurons per fish, transplant efficiency, cells transplanted per fish.

**File name:** Video 1

**Description:** Time-lapse illustrating the motility of a single transplanted zf-iMG (WTC-mEGFP-AAVS1-cl6; green). Microscopy data acquired using Bruker Luxendo TruLive3D Imager.

**File name:** Video 2

**Description:** Time-lapse illustrating the collective motility of transplanted zf-iMG (WTC-mEGFP-AAVS1-cl6; green). Microscopy data acquired using Bruker Luxendo TruLive3D Imager

**File name:** Video 3

**Description:** Time-lapse illustrating branch dynamics of transplanted zf-iMG (WTC-mEGFP-AAVS1-cl6; green). Microscopy data acquired using Andor Dragonfly 200 Sona spinning-disc microscope.

**File name:** Video 4

**Description:** Time-lapse illustrating transient contacts between zf-iMG (WTC-mEGFP-AAVS1-cl6; green) and zebrafish apoptotic neurons (Tg(nbt:dLexPR-LexOP:secA5-BFP); orange). Microscopy data acquired using Bruker Luxendo TruLive3D Imager

**File name:** Video 5

**Description:** Time-lapse illustrating coexistence of human-like microglia (WTC-mEGFP-AAVS1-cl6; green) and endogenous zebrafish microglial cells (Tg(fms:Gal4,UAS:nfsB-mCherry); purple). Microscopy data acquired using Andor Dragonfly 200 Sona spinning-disc microscope.

**File name:** Video 6

**Description:** Time-lapse illustrating engulfment of an apoptotic zebrafish neurons (Tg(nbt:dLexPR-LexOP:secA5-BFP); orange) by a zf-iMG (WTC-mEGFP-AAVS1-cl6; green)

**File name:** Video 7

**Description:** Time-lapse illustrating multiple attempts of engulfment of an apoptotic zebrafish neurons (Tg(nbt:dLexPR-LexOP:secA5-BFP); orange) by a zf-iMG (WTC-mEGFP-AAVS1-cl6; green)

**File name:** Video 8

**Description:** Time-lapse illustrating zf-iMG (WTC-mEGFP-AAVS1-cl6; green) response to brain laser ablation. Microscopy data acquired using Andor Dragonfly 200 Sona spinning-disc microscope

**File name:** Video 9

**Description:** Time-lapse illustrating convergence of engulfed apoptotic material (Tg(nbt:dLexPR-LexOP:secA5-BFP); orange) inside a zf-iMG (WTC-mEGFP-AAVS1-cl6; green). Microscopy data acquired using Andor Dragonfly 200 Sona spinning-disc microscope

**File name:** Video 10

**Description:** 3D ultrastructural visualization of hIPSC-derived microglia in brain organoids. Microscopy data acquired using Leica THUNDER microscope

**File name:** Video 11

**Description:** Time-lapse illustrating TREM2 mutant zh-iMG (WTC-mEGFP-AAVS1-cl6; blue) maintaining dynamic surveillance in vivo. Endogenous apoptotic neurons are marked in orange (Tg(nbt:dLexPR-LexOP:secA5-BFP)). Microscopy data acquired using Andor Dragonfly 200 Sona spinning-disc microscope
