# Supplementary material for: A scalable human-zebrafish xenotransplantation model reveals gastrosome-mediated processing of dying neurons by human microglia
Source: Commun Biol. 2026 Apr 9;9:785. doi: 10.1038/s42003-026-09948-6 (PMC13250125; doi:10.1038/s42003-026-09948-6)
Supplement: Supplementary file 15 — Reporting Summary [file 42003_2026_9948_MOESM15_ESM.pdf]

nature portfolio

Corresponding author(s): *Double-anonymous peer review submissions: write DAPR and your manuscript number here instead of author names.*

Last updated by author(s): *YYYY-MM-DD*

Reporting Summary

Nature Portfolio wishes to improve the reproducibility of the work that we publish. This form provides structure for consistency and transparency in reporting. For further information on Nature Portfolio policies, see our Editorial Policies and the Editorial Policy Checklist.

Statistics

For all statistical analyses, confirm that the following items are present in the figure legend, table legend, main text, or Methods section.

n/a	Confirmed	
☐	☒	The exact sample size (*n*) for each experimental group/condition, given as a discrete number and unit of measurement
☐	☒	A statement on whether measurements were taken from distinct samples or whether the same sample was measured repeatedly
☐	☒	The statistical test(s) used AND whether they are one- or two-sided<br>*Only common tests should be described solely by name; describe more complex techniques in the Methods section.*
☒	☐	A description of all covariates tested
☐	☒	A description of any assumptions or corrections, such as tests of normality and adjustment for multiple comparisons
☐	☒	A full description of the statistical parameters including central tendency (e.g. means) or other basic estimates (e.g. regression coefficient) AND variation (e.g. standard deviation) or associated estimates of uncertainty (e.g. confidence intervals)
☐	☒	For null hypothesis testing, the test statistic (e.g. *F*, *t*, *r*) with confidence intervals, effect sizes, degrees of freedom and *P* value noted<br>*Give P values as exact values whenever suitable.*
☒	☐	For Bayesian analysis, information on the choice of priors and Markov chain Monte Carlo settings
☒	☐	For hierarchical and complex designs, identification of the appropriate level for tests and full reporting of outcomes
☒	☐	Estimates of effect sizes (e.g. Cohen's *d*, Pearson's *r*), indicating how they were calculated

Our web collection on statistics for biologists contains articles on many of the points above.

Software and code

Policy information about availability of computer code

Data collection	Fusionv2.3(Andor);ZEN v2.3(Zeiss);InCellAnalyzer2500HSv7.1(Cytiva);AmershamImage600v1.2(Cytiva);MAPS v3(ThermoFisher Scientific), CFX96 Real-Time System v5.0 (Bio-Rad)
Data analysis	FIJI- ImageJ v2.0 with Bio-Formats, BigDataProcesso12 and MTrackJ plugins; Imaris v9.8 (Bitplane); Python v3.8 (Python Software Foundation); Microsoft Excel v16.83 (Microsoft), Compound Discoverer v3.3 (Thermo Fisher Scientific)

For manuscripts utilizing custom algorithms or software that are central to the research but not yet described in published literature, software must be made available to editors and reviewers. We strongly encourage code deposition in a community repository (e.g. GitHub). See the Nature Portfolio guidelines for submitting code & software for further information.

Data

Policy information about availability of data

All manuscripts must include a data availability statement. This statement should provide the following information, where applicable:

- Accession codes, unique identifiers, or web links for publicly available datasets
- A description of any restrictions on data availability
- For clinical datasets or third party data, please ensure that the statement adheres to our policy

The data and material supporting the findings of this study are available from the corresponding author upon request. Source data are provided in the paper.

Research involving human participants, their data, or biological material

Policy information about studies with human participants or human data. See also policy information about sex, gender (identity/presentation), and sexual orientation and race, ethnicity and racism.

Reporting on sex and gender	N/A
Reporting on race, ethnicity, or other socially relevant groupings	N/A
Population characteristics	N/A
Recruitment	N/A
Ethics oversight	N/A

Note that full information on the approval of the study protocol must also be provided in the manuscript.

Field-specific reporting

Please select the one below that is the best fit for your research. If you are not sure, read the appropriate sections before making your selection.

☒ Life sciences ☐ Behavioural & social sciences ☐ Ecological, evolutionary & environmental sciences

For a reference copy of the document with all sections, see nature.com/documents/nr-reporting-summary-flat.pdf

Life sciences study design

All studies must disclose on these points even when the disclosure is negative.

Sample size	No statistical method was used to predetermine the sample size. Based on the large sample size and high reproducibility of the results, the sample slze was considered sufficient.
Data exclusions	No data exclusiona were made.
Replication	The experimental findings were reproduced In multiple independent experiments. In all zebrafish experiments, several animals were analyzed per group (indlcated by N). All cell experiments were performed in three independent experiments. The numbers of replicates are noted in each figure legend. Data were consistent across repetitions.
Randomization	For animal experiments, we randomized the order of data acquisition across different conditions. For in vitro experiments, randomization is not relevant since cells come in milllons of populations and are automatically randomized and seeded to different wells for treatment.
Blinding	Investigators were not blinded. The data were analyzed using detection, tracking and analysis conditions that were maintained constant between experimental conditions.

Reporting for specific materials, systems and methods

We require information from authors about some types of materials, experimental systems and methods used in many studies. Here, indicate whether each material, system or method listed is relevant to your study. If you are not sure if a list item applies to your research, read the appropriate section before selecting a response.

Materials & experimental systems

n/a	Involved in the study	
☐	☒	Antibodies
☐	☒	Eukaryotic cell lines
☒	☐	Palaeontology and archaeology
☐	☒	Animals and other organisms
☒	☐	Clinical data
☒	☐	Dual use research of concern
☒	☐	Plants

Methods

n/a	Involved in the study	
☒	☐	ChIP-seq
☐	☒	Flow cytometry
☒	☐	MRI-based neuroimaging

Antibodies

Antibodies used	All a ntibodies used in this study were commercially availa ble a ntibodies. Anti human CD43 (STEMCELL Technologles 6008542, 1:100), Anti-human CD45 (STEMCELLTechnologies 6001842.1, 1:100), Anti-human CD34 (ThermoFisher Scientiflc, CD34-581-04, 1:100), Anti-human CD45 (STElVCELLTechnologies, 6001842.1; 1:200) Anti-human CD11b (STEMCELLTechnologies, 60040PE.1, 1;100) Antihuman CD14(STEMCELLTechnologies, 60004A2.1, 1:200) Anti-human TREM2 (R&D Systems, AF1828, I:2O (FACS), 1oug/ml (lHC) Anti-human pU.1 (Cell Signaling, 22665, I:4O (FACS) 1:1000 (lHC)) Anti-human P2Y12 (Merck, HPA014518, 1:25) Anti-human lBAl (Syna ptic Systems , 234OO3, 1:250 (FACS) 1:1000 (l HC)) Anti-goat A F546 (ThermoFisher Scientific, A-11056, 1:500) Anti-ra bbit AF568 (Molecular Probes, 411011, 1:500).
Validation	All antibodies are commercially available and have been tested and validated by their manufacturer for use ln FACS and lHC. Antihuman CD43 (STEMCELL Technologies 60085AZ, validation can be found https://www.stemcell.com/products/anti-human-cd43 antibody-clone-cd43-1097.html), Anti-human CD45 (STEMCELL Technologies 60018A2.1, validatlon can be found https:// www.stemcell.com/products/anti-human-cd45-antibody-clone-hi30.html), Antl-human CD34 (ThermoFisher Screntific, CD34-581 04, vall dation ca n be fou nd https://www.thermofisher.com/a nti body/product/CD34-Anti body-clone-581-Monoclona l/CD34-581-04), Anti-human CD45 (STEMCELL Technologies, 60018A2.1; validation can be found https://www.stemcell.com/products/anti-humancd45-antibody-clone-hi30.html) Anti-human CD11b (STEMCELL Technologies, 60040PE.1, validation can be found https:// www.stemcell.com/products/anti-human-cd11b-antibody-clone-icrf44,html) Anti-humanCD14(STEMCELLTechnologies,60004AZ.1,, validation can be found https://cdn,stemcell.com/mediafiles/p1s/27679-PlS_1_0_0.pdf) Anti-human TREM2 (R&D Systems, AF1828, 1:20 (FACS), validation can be found https://www.rndsystems.com/products/human-trem2-antibody_af1828) Anti-human pU,1 (Cell Signaling, 22665, validation can be found https://www.cellsignal.com/products/primary-antibodies/pu-1-antibody/2266? srsltid=AfmBOoqZzPytU3XRvBvMndDFMQwDYG69-NsQe6sUfAOkSfCJfe_rplVj) Anti-human P2Y12 (lvlerck, HPAO14518, validation can be found https://www.merckmillipore.com/BA/en/product/sigma/hpa014518) Anti-human lBAl (Synaptic Systems, 234003, validatlon can be found https://sysy.com/iba1) Anti-goat AF546 (ThermoFisher Scientific, A-11056, validation can be found https:// www.thermofisher.com/antibodV/product/Donkey anti-Goat-lgG-H-L-Cross-Adsorbed-Secondary-Antibody-Polyclonal/A-11056) Anti-rabbit AF568 (Molecular Probes, A11011, validation can be found https://www.thermofisher.com/antibody/product/Goat-anti-Ra bbit-lgG-H-L-Cross-Adsorbed-Seconda ry-Anti body-Polyclona l/A-1101 thttps://www.thermoflsher.com/a ntl body/product/ Goatanti-Rabbit-lgG-H-L-Cross-Adsorbed-Secondary Antibody-Polyclonal/A-11011).

Eukaryotic cell lines

Policy information about cell lines and Sex and Gender in Research

Cell line source(s)	TC mEGFP-Safeharborlocus(AAVS1)-cl6(mono-allellc tag) (Allen Institute for Cell Science, AICS-0036-006; from a male donor)j WTC-mTagRFPT-CAAX-Safeharborlocus(AAVS1)-c191 (mono-allelic tag; Allen Institute for Cell Science AICS-0054-09L; from a male donor) Cent 3-6 and Clue4-7(A detailed description of all reprogrammed iPSCs can be found in (Schafer et al, Nat Neuroscience 2019) . Additional information was published in Marchetto et al., Mol. Psychiatry (20L6). The iPSC lines obtained were reprogrammed in the same facllity (5alk Institute for Biologlcal Studies, Laboratory of Genetics) and under the same conditlons. Briefly, fibroblasts were transduced with retrovlruses containing SOX2, OCT4, KLF4 and MYC to induce overexpresslon ofthese genes and were transferred to a co-culture system with murine embryonic fibroblasts. IPSC colonles were identified after around two weeks in this culture system, plated onto Matrigel-coated plates (BD Biosciences) and maintained in mTeSRl media (Stem Cell Technologres), Experiments were performed with 5 independent control lines (Clue, Cent, Cove, Chap and Cent) The above mentioned identifrers, accompanied by their respective clone lD, have also been used to allow sample identification in the deposited raw sequencing data.
Authentication	IPSC llnes were grown separately and authentication was performed through the use ofstandardized names and clonespecific unique iPSC lines were grown separately and authentication was performed through the use of standardized names and clone-specific unique identifiers.
Mycoplasma contamination	All cell lines tested negative for mycoplasma contamination
Commonly misidentified lines (See ICLAC register)	No commonly misidentified cell lines were used

Animals and other research organisms

Policy information about studies involving animals; ARRIVE guidelines recommended for reporting animal research, and Sex and Gender in Research

Laboratory animals	Zebrafish (Danio rerio) were raised, maintained, and bred according to the standard procedures. All experiments were performed on embryos younger than 5dpf. The following mutant and transgenic animals in the TUBINGEN and Golden background were used in this study: (irf8st95; Shiau, C. E. et al, 2015; ZDB-FISH-150901-4256); slc37a2NY007 (Vlllani et al, 2019; ZDB-FISH-191226-5); Tg(mpeg1:GFP-caax)(Villani et al, 2019; ZDB-TGCONSTRCT-I91,2I1--I]1; TgBAC(fms:Gal4,UAS:nfsB-mCherry) (Gray et al., 201,1,;ZDB-ALT-1,I0707-2); Tg(UAS:lyn-miRFP670) (this paper)
Wild animals	No wlld animals were used in this study.
Reporting on sex	Sex ofthe fish was not considered in this study, as in zebrafish sex differentiatlon takes place around 20-25dpf
Field-collected samples	No field collected samples were used in this study.

Ethics oversight	All experiments were performed on zebrafish embryos younger than 5dpl in accordance with the European Union Directive 2O1O/62/EU and local authorities (Kantonales VeterianämU Fishroom licence TVHa Nr. 178.)

Note that full information on the approval of the study protocol must also be provided in the manuscript.

Plants

Seed stocks	N/A
Novel plant genotypes	N/A
Authentication	N/A

Flow Cytometry

Plots

Confirm that:

☒ The axis labels state the marker and fluorochrome used (e.g. CD4-FITC).

☒ The axis scales are clearly visible. Include numbers along axes only for bottom left plot of group (a 'group' is an analysis of identical markers).

☒ All plots are contour plots with outliers or pseudocolor plots.

☒ A numerical value for number of cells or percentage (with statistics) is provided.

Methodology

Sample preparation	On day 12 ofiHPC differentiation, hematopoietic markers were tested via antibody staining and FACS analysis. 5*104 E 2*105 cells/sample were incubated with antibodies diluted in FACS Buffer (D-PBS without Mg++ and Ca++ with 2% FBS) for 3Omin at 4"C. The cells were washed twice with FACS buffer and resuspended in 300u1 sorting buffer (FACS buffer with 1mM EDTA and 25mlvl HEPES) for analysis. On day 24 of |MGL differentiation, microglial markers were tested via antibody staining and FACS analysis. 5*104 E 2*105 cells/sample were resuspended in 100 ;rl of FACS buffer and blocked with 10Oul of 1opg/ml CD32 (20min at 4"C). The samples were then incubated 45 min at 4"C with the primary antibodies (see list below), and then 20 min at room temperature with secondary antibodies. The cells were washed twice wlth FACS buffer and then resuspended in 300ul sorting buffer for analysrs. The samples were analyzed at a BD LSR ll Fortessa Analyzer and processed in FlowJo.
Instrument	BD LSR ll Fortessa Analyzer
Software	FlowJo
Cell population abundance	For flow cytometry analysis (measuring MFls for reporter assays), a similar number of cells were analyzed for each replicate as indicated in the Supplementary lnformation (5*104 E 2+105 cells/sample). Gates and histograms are shown for the populations analyzed and percentages for cell populations were indicated
Gating strategy	Gating strategies for flow cytometry analysls are shown ln the figures, including the respective isotype controls to determlne the gates. ln FlowJo, flow cytometry data (.FCS files) are analyzed through a stepwise gating strategy. First, forward and side scatter are used to exclude debris and select intact cells. Doublets are then removed using pulse geometry parameters to ensure single-cell events. Finally, fluorescence-based gates, guided by appropriate controls, are applied to identify and quantify specific cell populations.

☒ Tick this box to confirm that a figure exemplifying the gating strategy is provided in the Supplementary Information.
